# Identification of two new QTLs of maize (*Zea mays* L.) underlying kernel row number using the HNAU-NAM1 population

**DOI:** 10.1186/s12864-022-08793-1

**Published:** 2022-08-15

**Authors:** Xiaohong Fei, Yifei Wang, Yunxiao Zheng, Xiaomeng Shen, Lizhu E, Junqiang Ding, Jinsheng Lai, Weibin Song, Haiming Zhao

**Affiliations:** 1 State Key Laboratory of Plant Physiology and Biochemistry, China Agricultural University, Beijing, 100193 People’s Republic of China; 2 Department of Plant Genetics and Breeding, National Maize Improvement Center, China Agricultural University, Beijing, 100193 People’s Republic of China; 3 Longping Agriculture Science Co. Ltd, Beijing, 100004 People’s Republic of China; 4 State Key Laboratory of Wheat and Maize Crop Science and Center for Crop Genome Engineering, Henan Agricultural University, Zhengzhou, 450046 People’s Republic of China

**Keywords:** Maize, Kernel row number (KRN), Quantitative trait locus (QTL) mapping, Nested association mapping (NAM) population

## Abstract

**Background:**

Maize kernel row number (KRN) is one of the most important yield traits and has changed greatly during maize domestication and selection. Elucidating the genetic basis of KRN will be helpful to improve grain yield in maize.

**Results:**

Here, we measured KRN in four environments using a nested association mapping (NAM) population named HNAU-NAM1 with 1,617 recombinant inbred lines (RILs) that were derived from 12 maize inbred lines with a common parent, GEMS41. Then, five consensus quantitative trait loci (QTLs) distributing on four chromosomes were identified in at least three environments along with the best linear unbiased prediction (BLUP) values by the joint linkage mapping (JLM) method. These QTLs were further validated by the separate linkage mapping (SLM) and genome-wide association study (GWAS) methods. Three KRN genes cloned through the QTL assay were found in three of the five consensus QTLs, including *qKRN1.1*, *qKRN2.1* and *qKRN4.1*. Two new QTLs of KRN, *qKRN4.2* and *qKRN9.1,* were also identified. On the basis of public RNA-seq and genome annotation data, five genes highly expressed in ear tissue were considered candidate genes contributing to KRN.

**Conclusions:**

This study carried out a comprehensive analysis of the genetic architecture of KRN by using a new NAM population under multiple environments. The present results provide solid information for understanding the genetic components underlying KRN and candidate genes in *qKRN4.2* and *qKRN9.1*. Single-nucleotide polymorphisms (SNPs) closely linked to *qKRN4.2* and *qKRN9.1* could be used to improve inbred yield during molecular breeding in maize.

**Supplementary Information:**

The online version contains supplementary material available at 10.1186/s12864-022-08793-1.

## Introduction

Maize (*Zea mays* L.) is one of the most important cereal crops and plays important roles in food, animal feed and raw materials [1, 2]. Therefore, the inheritance of grain yield in maize has been a focus of agricultural scientists and plant breeders [3]. Kernel row number (KRN), which is highly heritable, is not only one of the most important yield components but also an important breeding target of maize [4]. In addition, KRN is a quantitative trait that appears to be genetically controlled by multiple genes, as more than 100 QTLs have been identified (http://www.maizegdb.org/) [5, 6]. Thus, it is of great significance to dissect the genetic architecture of the KRN.

To date, multiple genes involved in regulating maize inflorescence architecture and development that affect KRN have been identified, such as *THICK TASSEL DWARF1* (*TD1*), *FASCIATED EAR2* (*FEA2*), *RAMOSA1* (*RA1*), *RAMOSA2* (*RA2*) and *UNBRANCHED3* (*UB3)* [7–11]. Most of these genes have been isolated using inflorescence mutants. Almost all these mutants show sharply increased KRN but also negative effects on other related traits. For example, they have short ears [7, 8], a dwarf stature [12], and fasciated ear tips with unproductive kernels [10, 12]. Such negative effects have directly limited the application of these genes in maize yield improvement. Several studies have shown that KRN and yield can be increased to a certain extent without changing the main shape of the ear by creating weak allele mutants of maize ear meristem maintenance genes [13–15]. More recently, gene editing technology has been used to help manipulate the transcriptional activity of the corresponding genes at promoter regions, such as *CLE7* and *FCP1* [16]. This provides a flexible strategy of using KRN genes to improve maize yield.

Aside from the genes cloned from mutants, more natural variations controlling the KRN are widely distributed in the genome in the form of multiple genetic loci. Liu et al. [11] cloned *KRN4*, a major QTL with a 1.2-kb presence/absence variant (PAV) that regulated the expression of *UB3,* which encoded an SBP transcription factor that could participate in the regulation of meristem maintenance [11]. Wang et al. [17] identified and cloned another major QTL, *KRN1*, which corresponded to the AP2 domain-encoding gene *IDS1*/*TS6*. The results suggest that plants that produce more *IDS1*/*TS6* transcripts develop more spikelet pair meristems, resulting in more KRNs [17]. Recently, Chen et al. [18] identified the gene *KRN2* in a convergent selection region in cereal crops using a set of introgression lines constructed with maize Mo17 and teosinte. *KRN2* encoded a WD40 protein and functioned synergistically with a gene of unknown function, DUF1644. Knockout of *KRN2* alleles in maize and rice increased the yield to 10% and 8% in the investigated background, respectively [18]. The above studies show that the regulation of maize KRN involves pathways such as the CLAVATA-WUSCHEL, RAMOSA, regulation of auxin, cytokinin, and other metabolic pathways, reflecting the genetic complexity of KRN. Although reliable genetic loci of KRN can be obtained by QTL mapping assays, the large size and complexity of the maize genome has slowed the progress on QTL cloning works [19].

Traditionally, QTL mapping studies have been performed with linkage mapping strategies using segregating populations derived from biparental crosses, such as F_2_ populations, recombinant inbred lines (RILs), double-haploid populations (DHs), and single-segment substitution lines (SSSLs) [20–23]. Genome-wide association studies (GWASs) benefit from abundant diversity, enabling the locations of identified QTLs to be inferred with a high resolution; however, the inherent population structure and presence of rare variants in natural populations reduce GWAS statistical power [19]. Thus, several advanced populations have been developed for and introduced into GWASs, such as nested association mapping (NAM) [24], multiparent advanced generation intercross (MAGIC) [25], and complete-diallel design plus unbalanced breeding-like intercross (CUBIC) populations [26]. A NAM population simultaneously exploits the advantages of both linkage and association mapping and has advantages such as the reduced marker density requirement, increased allele richness, increased mapping resolution, and increased statistical power for QTL mapping [27]. Among crops, NAM populations have been gradually applied for barley, sorghum, wheat, rice, and especially maize [28–32]. Through these mapping approaches, a large number of QTLs for complex traits such as flowering time, biological stress resistance and kernel composition have been identified in maize [33–35]. The NAM population in this study was developed in a former work and reflected a high QTL identification power in the maize plant architecture [36].

In this study, we measured the KRN in the HNAU-NAM1 population with 1,617 RILs in four environments. Linkage mapping and GWASs were performed together to detect the QTLs underlying KRN. After integrating all the mapping results, we identified five consensus QTLs located on chromosomes 1, 2, 4, and 9 that explained 4.4%-18.3% of the phenotypic variation. Moreover, all the three KRN genes cloned in previous studies were also identified in the QTL intervals of our present study. In addition, we found two new dependable QTLs, *qKRN4.2* and *qKRN9.1*. Further analysis of the two QTLs revealed that five genes were speculated to be candidate KRN genes underlying *qKRN4.2* and *qKRN9.1.*

## Results

### Phenotypic evaluation

The 13 parents of HNAU-NAM1 population showed a large phenotypic variation in KRN. As shown in Table S1, the KRN of all parents varied from 9.3 in CML360 to 19.5 in DAN598. The KRN of HNAU-NAM1 ranged from 10.7–19.0, 11.0–19.0, 10.0–19.0, and 9.0–18.8 across the four environments (Table 1 and Table S2). This wide variation in KRN in each of the environments was beneficial for genetic architecture dissection. The KRN decreased from Sanya2020 (15.1 ± 1.6), Sanya2021 (14.6 ± 1.7), and Changge2020 (14.6 ± 1.5) to Beijing2021 (14.4 ± 1.4), as shown in Table 1. Among 12 subpopulations, the KRN varied from 13.9 ± 1.4 in Subpop CML304 to 15.5 ± 1.1 in Subpop DAN598 (Table S3).

**Table 1:** ANOVA, broad-sense heritability analysis, and descriptive statistics for HNAU-NAM1

Trait	Variation source	*F* value^a^	Broad-sense heritability	Descriptive statistics		
				**Environment**	**Range**	**Mean ± SD**^**b**^
Kernel Row Number	Genotype	4.512^a^	0.82	Changge2020	10.7–19.0	14.6 ± 1.5
				Sanya2020	11.0–19.0	15.1 ± 1.6
	Environment	83.299^a^				
				Sanya2021	10.0–19.0	14.6 ± 1.7
	Replication	0.706		Beijing2021	9.0–18.8	14.4 ± 1.4

^a^, *P* ≤ 0.01

^b^SD stands for standard deviation

**Fig. 1:**
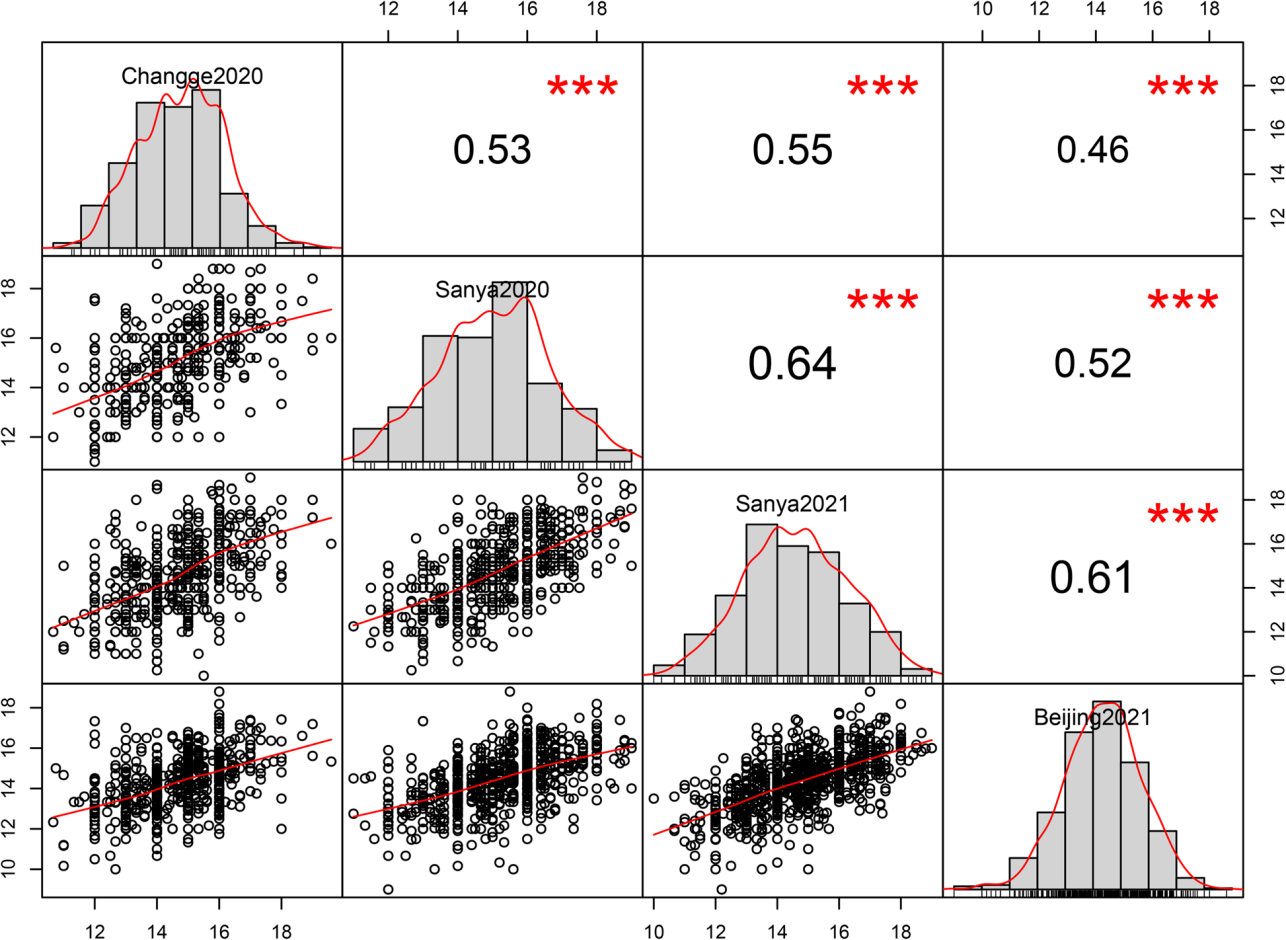
Correlation of the KRN phenotypes among Changge2020, Sanya2020, Sanya2021, and Beijing2021. Frequency distribution diagrams of KRN in the four environments are plotted, and the correlation coefficient between each pair of environments is shown. ***, *P* ≤ 0.001

As shown in Table 1, KRN exhibited high inheritance with a broad-sense heritability (*H*^*2*^) of 0.82, consistent with the suggestions of previous studies [37]. Analysis of variance (ANOVA) of HNAU-NAM1 showed significant variations for genotypes and environments (Table 1). In addition, KRNs were modestly correlated (*r* = 0.46–0.64) among the four environments (Fig. 1). The results indicated that the main proportion of the phenotypic variations in KRN were derived from genetic factors; at the same time, however, KRN was affected by the environment, mainly in Sanya2020. Therefore, it is necessary to perform QTL mapping with KRN data in different environments and to use best linear unbiased prediction (BLUP) values to comprehensively identify and assess the genetic effects of QTL regions.

### Linkage map construction of HNAU-NAM1

**Fig. 2:**
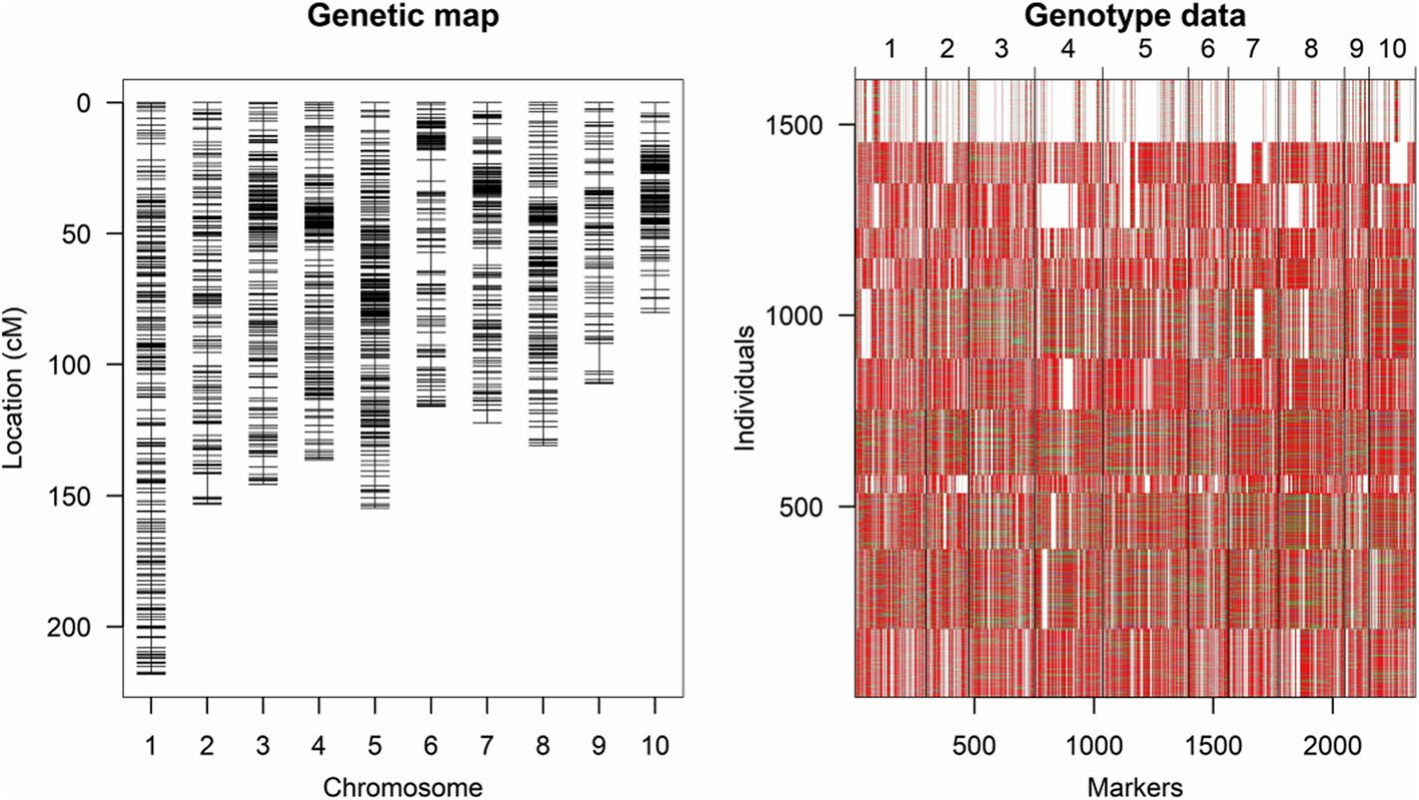
Joint linkage map of the HNAU-NAM1 population. Red: homozygous genotype of GEMS41; green: homozygous genotype of other parents; blue: heterozygous genotype. The ordinate represents the number of RILs

HNAU-NAM1 was constructed from crosses between the maize inbred line GEMS41 and 12 other inbred lines. These 12 biparental families contain 47–209 different backcross population-derived lines consisted of a total of 1,617 RILs. Detailed information about HNAU-NAM1 is given in Table S3. The number of segregating single-nucleotide polymorphism (SNP) markers ranged from 723 to 3,500 per subpopulation (Table 2 and Fig. 2). In addition, 77.8% of genotypes were homozygous for GEMS41, 17.0% were homozygous for the other parents, and 5.2% were heterozygous throughout the whole genome (Table 2).

**Table 2:** HNAU-NAM1 linkage map statistics

Subpopulation	Generation	No. RILs^a^	No. markers	Length (cM)	No. crossovers	G/P^b^	GEMS41 (%)	Heterozygous (%)	Other parent (%)
Subpop CIMBL29	BC_2_F_4_	179	2280	2093	2039	1.00	84.8	3.5	11.7
Subpop CIMBL83	BC_1_F_4_	209	3500	1382	4231	0.66	71.3	7.2	21.5
Subpop CML304	BC_1_F_4_	147	3433	1224	2715	0.58	71.1	6.3	22.6
Subpop CML360	BC_2_F_4_	47	3047	993	611	0.47	86.5	2.8	10.7
Subpop CML454	BC_1_F_4_	171	3465	1291	3309	0.61	70.9	7.6	21.5
Subpop CML470	BC_2_F_4_	133	3365	1196	1557	0.57	85.5	3.9	10.7
Subpop CML486	BC_1_F_4_	183	3336	1215	3475	0.58	71.4	6.8	21.8
Subpop CML496	BC_2_F_4_	80	3299	1470	1297	0.70	86.3	1.6	12.1
Subpop DAN598	BC_2_F_4_	79	3404	1362	1174	0.65	86.5	2.0	11.5
Subpop K22	BC_2_F_4_	117	3238	1248	1585	0.59	87.9	1.6	10.5
Subpop P178	BC_2_F_4_	108	3382	1408	1577	0.67	86.1	2.9	10.9
Subpop TY1	BC_1_F_4_	164	723	1270	1967	0.60	71.1	7.3	21.7
Composite	-	1617	2345	1376	25,537	0.66	77.8	5.2	17.0

^a^No, number of; RILs, recombinant inbred lines

^b^G/P stands for the ratio of genetic distance to physical distance relative to the maize B73 reference genome v4

We next constructed a genetic linkage map with a total of 2,345 markers that showed polymorphisms in at least 6 subpopulations. A joint linkage map with a length of 1,376 cM and 25,537 crossovers was constructed using the “R/qtl” package in R software. We examined the relationship between genetic distance based on the composite genetic map and physical distance according to the chromosome lengths of the B73 reference genome v4 (G/P). The mean value of G/P of each chromosome was 0.66 (Table 2).

Separate linkage maps were constructed for the 12 subpopulations, with an average length of 1,346 ± 254.35 cM (ranging from 993 cM in Subpop CML360 to 2,093 cM in Subpop CIMBL29) using the same method mentioned above (Table S4 and Fig. S1). On average, each linkage map comprised 3,039 SNP markers, and the average G/P among the 12 RIL subpopulations of HNAU-NAM1 varied from 0.47 to 1.00.

### Genetic dissection of KRN

**Fig. 3:**
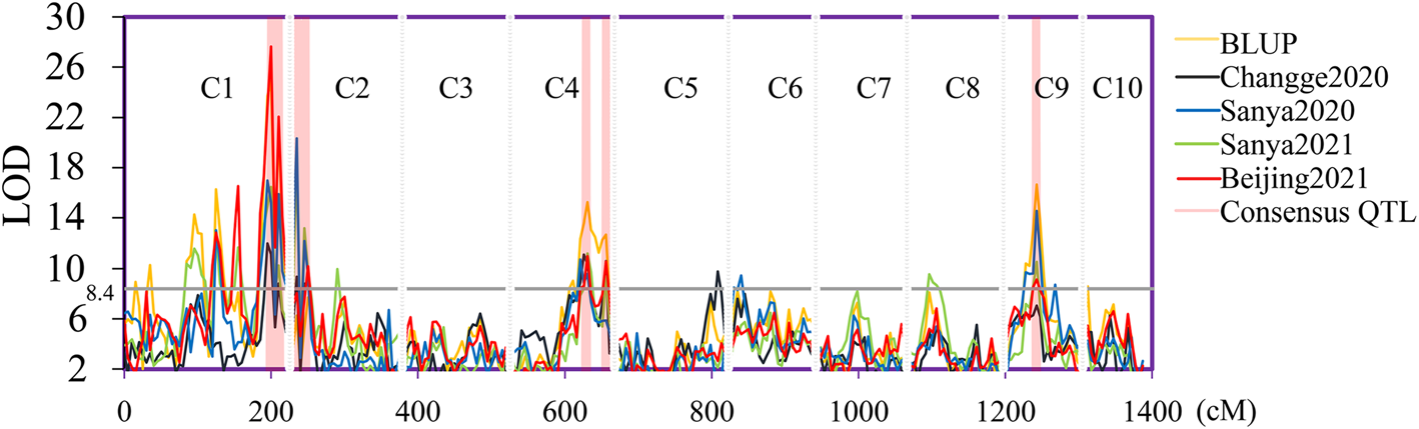
QTLs associated with KRN under different environments in the HNAU-NAM1 population as determined by the JLM method. C1-C10 represent chromosomes 1–10, respectively. The abscissa represents the genetic distance of the HNAU-NAM1 population. cM represents centimorgan

In joint-linkage mapping (JLM) analysis, 6–12 QTLs were identified in each environment using the BLUP value, which explained 4.4%-18.3% of the phenotypic variance (Fig. 3 and Table S5). Importantly, five consensus QTLs were identified in at least three environments according to the overlap of the QTL physical support intervals (Table 3). These QTLs were located on chromosomes 1, 2, 4, 4 and 9 and explained 8.2%-18.3%, 5.8%-10.4%, 5.6%-11.0%, 4.4%-9.5% and 4.8%-13.5% of the phenotypic variation in each environment, respectively.

**Table 3:** Consensus QTLs identified under different environments by JLM

No	QTL	Environment	Chromosome	QTL position (cM)	1.5-LOD support interval (B73_v4) (Mb)^a^	Peak LOD^b^	PVE (%)^c^
1	*qKRN1.1*	BLUP	1	200.0	282.9–303.1	25.0	18.3
		Changge2020	1	195.0	282.3–303.1	12.0	8.2
		Sanya2020	1	195.0	282.9–303.1	17.0	14.0
		Sanya2021	1	200.0	286.2–303.1	16.5	10.0
		Beijing2021	1	200.0	286.2–303.1	27.6	14.9
2	*qKRN2.1*	Changge2020	2	25.0	2.8–18.0	8.9	10.0
		Sanya2020	2	20.0	2.8–13.6	12.2	5.8
		Sanya2021	2	20.0	2.8–13.6	13.2	8.8
		Beijing2021	2	25.0	9.7–18.0	10.2	10.4
3	*qKRN4.1*	BLUP	4	105.7	196.5–214.5	15.3	11.0
		Changge2020	4	100.7	191.5–213.8	11.1	8.0
		Sanya2020	4	95.7	187–213.8	10.7	8.0
		Sanya2021	4	103.7	195.2–207.5	11.2	7.0
		Beijing2021	4	105.7	199.8–214.4	11.0	5.6
4	*qKRN4.2*	BLUP	4	129.7	238.5–243.4	12.7	9.5
		Changge2020	4	130.7	238.5–243.4	10.6	7.7
		Sanya2021	4	130.7	240.6–243.6	9.4	4.4
		Beijing2021	4	130.7	240.7–243.7	10.6	6.7
5	*qKRN9.1*	BLUP	9	45.6	101.7–110.2	16.7	13.5
		Sanya2020	9	45.6	101.7–111.8	14.6	11.7
		Sanya2021	9	43.6	97.6–110.2	10.5	5.1
		Beijing2021	9	45.6	97.6–110.2	9.1	4.8

^a^The 1.5-LOD support interval (B73_v4) (Mb) is based on the B73 reference genome v4

^b^LOD means logarithm of odds

^c^PVE (%) indicates the percentage of phenotypic variation explained by the QTL

To validate the reliability of the five consensus QTLs, we performed separate linkage mapping (SLM) and GWAS (Tables S6 and S7). We found that the five consensus QTLs could be repeatedly identified by the SLM method. First, *qKRN1.1* overlapped with 8 QTLs, which were identified in three subpopulations by the SLM method (SLM QTLs). Next, *qKRN2.1* overlapped with 6 SLM QTLs that were identified in four subpopulations. Then, *qKRN4.1* overlapped with 18 SLM QTLs that were identified in seven subpopulations. Furthermore, *qKRN4.2* overlapped with 7 SLM QTLs that were identified in four subpopulations. Lastly, *qKRN9.1* overlapped with 6 SLM QTLs that were identified in three subpopulations.

At the same time, we found that six SNPs that were significantly associated with KRN were located within four consensus QTL intervals. Among them, one SNP chr1_286427302 explained 2.14% and 0.49% of the phenotypic variation using Beijing and the BLUP value, respectively, which located within the *qKRN1.1* interval. Next, three SNPs, chr4_203363685, chr4_203327066 and chr4_203495247 were identified in Changge2020 and Beijing2021 using the BLUP value; these SNPs explained 7.36%, 4.85% and 7.18% of the phenotypic variation, respectively. We found that the flanking regions 500 kb upstream and 500 kb downstream of the three SNPs overlapped, and all were located within the *qKRN4.1* interval. Then, one SNP explaining 4.36% of the phenotypic variation, chr4_242133004l, was identified in Sanya2021. This SNP was located within the *qKRN4.2* interval. In addition, one SNP explaining 1.56% of the phenotypic variation, chr9_102720434, was identified using the BLUP value and was located within the *qKRN9.1* interval. These results provide reliable evidence of the five consensus QTLs.

### Identification of candidate genes underlying the two new dependable QTLs

Before the current study, only three KRN genes had been cloned through the QTL approach, namely, *KRN4* (*UB3*) [12], *KRN1* (*IDS1/TS6*) [17], and *KRN2* (a WD40-domain encoding gene) [18]. In this study, all these three KRN genes were identified in three of the five consensus QTL intervals. In addition, we also found two new dependable QTLs, *qKRN4.2* and *qKRN9.1*, which were repeatedly verified by SLM and GWAS methods. *qKRN4.2* and *qKRN9.1* explained 4.4%-9.5% and 4.8%-13.5% of the phenotypic variation in each environment. To further predict the candidate genes for KRN, we used the published RNA-seq datasets from 7 tissues [38] of all main plant organs across the whole development stages to profile the expression of genes annotated in the *qKRN4.2* and *qKRN9.1* regions with the B73 reference genome v4. As shown in Fig. S2, 46 and 38 genes were specifically expressed in the ear and tassel, respectively, relative to other tissues*.* Among them, we found some putative genes might controlling inflorescence development based on the functional annotations of their homologous genes. Thus, three candidate genes (*Zm00001d053756*, *Zm00001d053775*, encoding squamosa promoter binding protein; *Zm00001d053819*, encoding auxin response factor) and two candidate genes (*Zm00001d046783*, encoding GRAS family transcription factor; *Zm00001d046930*, encoding WD40 repeat-like superfamily protein) potentially associated with KRN were deduced in the two QTL regions, respectively (Table 4). We found that the expression of spike was relatively high in all tissues (Fig. S2). Furtherly, we checked gene expression level in the developing immature ear tissues. *Zm00001d053756*, *Zm00001d053775*, *Zm00001d046783*, and *Zm00001d046930* expressed relatively higher in 0.2–0.6 mm ear and decreased after 1 mm, according to the ear RNA-seq data of maize LH244 (Table S8, the raw data has not yet been published). The suppressed bract (SB) and spikelet pair meristem (SPM) initiated at 0.2 to 0.6 mm stage, indicating that these candidate genes might involve in the emergent of SPM, and further affected the KRN.

**Table 4:** Functional annotation of potential candidate genes

Number	QTL	Chromosome	Gene ID	Function annotation
1	*qKRN4.2*	4	*Zm00001d053756*	Squamosa promoter binding protein
2	*qKRN4.2*	4	*Zm00001d053775*	Squamosa promoter binding protein
3	*qKRN4.2*	4	*Zm00001d053819*	Auxin response factor
4	*qKRN9.1*	9	*Zm00001d046783*	GRAS family transcription factor
5	*qKRN9.1*	9	*Zm00001d046930*	WD40 repeat-like superfamily protein

## Discussion

### Characteristics and comparison of the three QTL positioning methods

The use of NAM populations becomes progressively a regular practice in crop genetics and crop breeding. NAM was constructed to enable high power and high resolution through joint linkage-association analysis [27]. In this study, we used the JLM method to perform a comprehensive genetic analysis of KRN in the maize HNAU-NAM1 population composed of 12 BC_1_F_4_/BC_2_F_4_ families. In maize HNAU-NAM1, a total of 47 QTLs associated with maize KRN in the four environments and using the BLUP value were identified. Moreover, five consensus QTLs were identified in at least three environments. To further verify the mapping results with the JLM method, we used two different methods to map QTLs and compare them with the five consensus QTLs. (1) SLM method. In the SLM mapping method, separate genetic linkage maps of 12 subpopulations were constructed, ranging in length from 993 to 2,093 cM (Table S4). A number of QTLs associated with the maize KRN were localized throughout almost all 10 chromosomes in maize by using these genetic maps. The phenotypic variation explained by QTLs (PVE) varied in different subpopulations, possibly because there were too few RILs in Subpop CML360, resulting in overestimation of the effect. If this subpopulation was removed, the PVE of each QTL ranged from 4.9% to 22.4%. In addition, some SLM QTLs overlapped with the five consensus QTLs located by the JLM method. (2) GWAS method. In the GWAS mapping method, a number of SNPs significantly associated with the maize KRN were identified. Among them, 6 SNPs significantly associated with KRN were found to be located within four consensus QTLs from the JLM method. However, we found that there was no SNP significantly associated with KRN located within the *qKRN4.1* interval. This may have been due to the lack of functional gene variation. In addition, one candidate gene (*Zm00001d053819*) was located in the QTL supporting regions of SNP chr4_242133004, providing evidence for screening of candidate genes potentially associated with KRN. In summary, these results indicate the reliability of the consensus QTLs and candidate genes.

### Characteristics of the consensus QTLs compared to those in former studies

Previous studies have found that many KRN QTLs are clustered in hotspots across the genome. Calderón et al. [39] mapped a region on the long arm of chromosome 1 containing the QTL *KRN1.4* for KRN, and this QTL had a 1.5-logarithm of odds (LOD) confidence interval of 203 kb. Wang et al. [17] finely mapped *KRN1* to a 6.6-kb genomic region on chromosome 1 based on the maize B73 reference genome v4. We noticed that *KRN1* was very close to *KRN1.4*, but *KRN1* was consistent with *KRN1.4*. In this study, one consensus QTL, *qKRN1.1,* was located on chromosome 1, which included the *KRN1.4* and *KRN1* intervals. Another major QTL, *KRN4*, was identified by combining linkage mapping and association analysis [40, 41]. Liu et al. [11] isolated *KRN4* by positional cloning. *qKRNW4,* a new KRN QTL, was narrowed down to a nearly 700-kb interval [42] located nearly 1 Mb away from *KRN4* [11]. Similarly, one consensus QTL in this study, *qKRN4.1*, was located on chromosome 4, which included the *KRN4* and *qKRNW4* intervals. However, some clustered QTLs were not identified in this study, such as *qKRN5a* and *qKRN5b* [43]. In addition, several QTLs containing a single KRN gene with large effects also overlapped in our results. For example, one consensus QTL *qKRN2.1* in this study included the *qKRN2* interval [18, 44]. However, some major QTLs were not identified in HNAU-NAM1, such as *qKRN5.04* and *qKRN8* [45, 46]. This may be because HNAU-NAM1 lacked variations in these functional loci.

Marker-assisted selection (MAS) can aid in selection of breeding progeny carrying desirable alleles to achieve the purpose of crop improvement [47]. In this study, three consensus QTLs, *qKRN4.1*, *qKRN4.2*, and *qKRN9.1*, tended to have negative additive effects on KRN (Table S6), indicating that the parent GEMS41 contributed the favorable allele. In contrast, two consensus QTLs, *qKRN1.1* and *qKRN2.1*, tended to have positive additive effects on KRN, indicating that other parents contributed the favorable allele. In summary, lines with favorable allele should be used for marker-assisted breeding and breeding improvement. So the genotype of the parent GEMS41 should be considered for *qKRN4.1*, *qKRN4.2*, and *qKRN9.1*, while other parents would provide the favorable allele at *qKRN1.1* and *qKRN2.1*. All these QTLs lay a foundation for marker-assisted breeding and breeding improvement.

### Putative genes involved in the regulation of KRN

We mainly focused on the five consensus QTLs simultaneously detected in at least three environments using the JLM method (Table 3). Interestingly, in these five QTL genomic regions, we identified three genes, namely, *Zm00001d034629* (*IDS1/TS6*), *Zm00001d002641* (a WD40-domain encoding gene), and *Zm00001d052890* (*UB3*), that are known to be involved in the regulation of KRN [12, 17, 18]. These most likely represent fundamental genes underlying KRN, and their location within consensus QTL regions strongly demonstrates the power and efficiency of our strategy to dissect the genetic basis of quantitative traits using HNAU-NAM1. Furthermore, we analysed the two dependable QTL regions *qKRN4.2* and *qKRN9.1* and identified potential candidate genes; for example, two squamosa promoter binding protein (SBP) family genes (*Zm00001d053756* and *Zm00001d053775*) and one WD40 protein gene (*Zm00001d046930*) were found to be located within the two QTL mapping regions. Importantly, Chuck et al. [12] showed that the genes *UB2* and *UB3* encoding the maize SBP transcription factors can affect maize yield by controlling the branching and differentiation of male and female inflorescences. Their findings further suggested that *ub2*/*ub3* double mutants display a decrease in tassel branch number and an increase in ear row number. Moreover, recent studies involving sequence analysis of *KRN2* have predicted that this gene encodes a cytoplasmic WD40 protein containing seven WD40 repeats [18]. Members of the WD40 family act as scaffolds for protein–protein interactions [48, 49] and have diverse functions in plants, including in development, metabolite biosynthesis, and immune responses [50, 51]. In addition, a gene (*Zm00001d053819*) that encodes an auxin response factor was found to be located in the *qKRN4.2* interval. Studies have shown that auxin biosynthesis and polar transport play an important role in maize ear development [52]. The ear length and kernel numbers of the maize functional deletion mutants *vanishing tassel 2* (*vt2*) and *sparse inflorescence1* (*spi1*) are decreased, and a similar phenotype is caused by a lack of auxin [53, 54]. Therefore, we speculate that this gene affects the formation of KRN by affecting auxin synthesis. Furthermore, a gene (*Zm00001d046783*) that encodes a GRAS family transcription factor was found in the *qKRN9.1* interval. GRAS proteins are plant-specific transcription factors that are involved in various developmental processes, including meristem maintenance, root radial patterning, light signalling, phytohormone signalling, and abiotic/biotic stress responses [55–58]. Cai et al. [59] isolated a GRAS transcription factor, ZmGRAS20, from the maize inbred line B73 based on transcriptome sequencing. Overexpression of *ZmGRAS20* led to the formation of a chalky region of ventral endosperm with decreased starch content and defective agronomic characteristics, including grain length, grain width, grain thickness, and 1000-grain weight, in transgenic seeds. Li et al. [60] identified a novel DELLA-like transcriptional regulator, ZmGRAS11, that positively regulates kernel size and kernel weight in maize. However, further fine-scale mapping and functional gene validation are required to confirm whether these candidate genes truly represent the causal agents underlying QTLs for KRN.

## Methods

### Plant materials and field trials

The maize NAM population, named HNAU-NAM1, contained 1,617 RILs that were derived from crosses of the common parent GEMS41 with each of 12 diverse inbred lines: CIMBL29, CIMBL83, CML304, CML360, CML454, CML470, CML486, CML496, DAN598, K22, P178 and TY1. The method of population construction was described in a previous study [36]. The 12 subpopulations were named Subpop CIMBL29, Subpop CIMBL83, Subpop CML304, Subpop CML360, Subpop CML454, Subpop CML470, Subpop CML486, Subpop CML496, Subpop DAN598, Subpop K22, Subpop P178, and Subpop TY1. The HNAU-NAM1 population was planted in four environments in China: Changge (34°13’N, 113°46’E) in 2020, Sanya (18°25’N, 109°50’E) in 2020 and 2021, and Beijing (39°54’N, 116°23’E) in 2021. Across all of the field trials, a randomized complete design was used. In every environment, the 12 subpopulations were randomly assigned to 12 plots with one or two replications. In every plot, each line of the corresponding subpopulation was planted in a row with 10 plants, with 0.25 m between plants and 0.60 m between rows. All lines of the HNAU-NAM1 population followed standard local field management practices using local maize tillage methods throughout the whole growth period. When maturity was reached, 5–7 ears of each line in every plot were harvested to calculate the KRN.

### Phenotypic analysis

Descriptive statistical analysis was performed with Microsoft Excel 2010. The broad-sense heritability (${\text{H}}^{2}$) for KRN was estimated using the formula ${\mathrm{H}}^{2}\text{=}{\delta }_{\text{g}}^{2}{/}({\delta }_{\text{g}}^{2}\text{+}{\delta }_{\text{e}}^{2}{/}{\text{n}}{)}$, where ${\delta }_{\text{g}}^{2}$ is the genetic variance, ${\delta }_{\text{e}}^{2}$ is the residual variance, and ${\text{n}}$ is the number of environments. The estimates of ${\delta }_{\text{g}}^{2}$ and ${\delta }_{\text{e}}^{2}$ were obtained with a mixed linear model treating genotype, environment and repetition as random effects. ANOVA was performed to evaluate the effects of genotype and environment on phenotypic variance in R [37]. Correlation analysis was performed in the R package “Performance Analytics”. Considering the effect of environmental variation on QTLs, the BLUP value was obtained for each line across all environments using the mixed linear model in the R package “lme4” [61] and adopted as a new phenotypic value in the following analyses.

### Linkage map construction and QTL identification

Polymorphic SNP markers were obtained from the MaizeSNP9.4 K BeadChip array, and severely segregated markers were removed in each subpopulation. Next, SNPs showing polymorphisms in at least 6 subpopulations were retained; thus, a total of 2,345 markers were retained to construct a joint genetic linkage map. The joint linkage map was constructed with Kosambi’s mapping function in a modified version of “R/qtl” software [62, 63]. Then, we conducted 1,000 permutations to determine the LOD significance threshold for QTLs at the *P* ≤ 0.05 level. To avoid overestimation of the number of QTLs, adjacent peaks within neighbouring genetic regions (≤ 10 cM) with the same effect directions were defined as a single QTL, as previously described [41]. For each QTL, a QTL support interval was defined as the 1.5-LOD drop position ranging from the QTL peak [64]. Finally, JLM was carried out using the NAM function of QTL IciMapping v4.2.53 software [65].

We performed SLM analysis using composite interval mapping in each RIL subpopulation to validate the JLM results. Genetic distances and LOD thresholds were calculated using the same method mentioned above. For convenience, an LOD = 3.0 was utilized as the global cut-off point.

### GWAS

To further verify the JLM results, we also performed a GWAS for HNAU-NAM1. After quality control, a total of 5,129 SNPs with a minor allele frequency (MAF) > 5%, missing rate < 20% and heterozygous rate < 50% were selected and used for the GWAS. The population structure was estimated using Admixture v1.3 software with the number of subpopulations (k) ranging from 1 to 15 [66], and the optimal number of subpopulations was approximately k = 12. Next, 5,129 SNPs were used to estimate the relative kinship by GCTA v1.92.2 software [67]. Then, a fixed and random effect (FarmCPU) model tool for GWAS in the R package “GAPIT” [68] was used on the KRN to test the statistical association between phenotypes and genotypes. Population structure and relative kinship were taken into account in the model to decrease spurious associations. After the GWAS, the criterion of the *P* value was set as 9.7e-6 (*P* ≤ 0.05/N, where N is the total number of genome-wide SNPs).

### QTL region identification

QTLs detected in at least three environments by the JLM method were identified as consensus QTL regions. Next, the QTL supporting regions identified in the JLM or SLM methods were obtained based on the physical coordinates of flanking markers. The mapping results from the GWAS combined the 500 kb upstream and downstream regions of the significant SNPs as the QTL supporting regions, as described by Zhao et al. [36]. Then, all the genes in the consensus QTL support interval were annotated according to the B73 reference genome v4 [69]. Raw data sets of RNA-Seq from different maize tissues were described from previous study [38]. RNA-seq reads were aligned to the maize B73 reference genome using Hisat2-2.2.1 [70]. We calculated the number of uniquely mapped reads for each gene model in the B73 FGS by parsing the alignment output files from Hisat2, and then normalized the resulting read counts by FPKM to measure the gene expression level.

## Supplementary Information

**Additional file 1:** **Table S1. **Phenotypic values of 13 parents of HNAU-NAM1 population. **Table S2. **Phenotype about the HNAU-NAM1 under four environments.** Table S3.** Detailed information about HNAU-NAM1. **Table S4.** Genetic map information for HNAU-NAM1 consisting of 12 RIL families. **Table S5.** JLM results for KRN in HNAU-NAM1. **Table S6.** SLM results for KRN in the 12 RIL families. **Table S7.** Candidate SNPs associated with KRN in HNAU-NAM1, as detected by GWAS analysis. **Table S8.** FPKM of five genes in different ear samples

**Additional file 2:** **Fig. S1 **Genetic linkage maps in the 12 subpopulations of HNAU-NAM1. Red: GEMS41 genotype; green: genotype of other parents; blue: heterozygote. The ordinate represents the number of RILs. **Fig. S2** Expression profiles of the genes located in the QTL regions *qKRN4.2* (A) and *qKRN9.1* (B). The expression values were collected from a public database (www.maizegdb.org) and normalized by the logarithm of fragments per kilobase of exon model per million mapped fragments (Log_2_(RPKM+1)). Each column represents a tissue, and the rows indicate genes expressed in the ear

## Data Availability

The main datasets supporting the conclusions of this article are included within the article and its additional file. The RNA-seq datasets used in this study are available in this reference (Chen J, Zeng B, Zhang M, Xie S, Wang G, Hauck A, Lai J. Dynamic transcriptome landscape of maize embryo and endosperm development. Plant Physiol. 2014; 166(1): 252–64. https://doi.org/10.1104/pp.114.240689). The sequence data in this article can be found in the NCBI Gene database (https://www.ncbi.nlm.nih.gov/gene/) under the following accession numbers: *TD1* (*Zm00001d014793*), *FEA2* (*Zm00001d051012*), *RA1* (*Zm00001d020430*), *RA2* (*Zm00001d039694*), *UB3* (*Zm00001d052890*), *KRN2* (*Zm00001d002641*) and *IDS1/TS6* (*Zm00001d034629*).

## Abbreviations

ANOVA: Analysis of variance
BLUP: Best linear unbiased prediction
CUBIC: Complete-diallel design plus unbalanced breeding-like intercross
GWAS: Genome-wide association study
*H*^*2*^: Broad-sense heritability
JLM: Joint linkage mapping
KRN: Kernel row number
MAF: Minor allele frequency
MAGIC: Multiparent advanced generation intercross
MAS: Marker-assisted selection
NAM: Nested association mapping population
PAV: Presence/absence variant
PVE: Phenotypic variation explained by QTLs
QTL: Quantitative trait locus
RIL: Recombinant inbred line
SB: Suppressed bract
SBP: Squamosa promoter binding protein
SLM: Separate linkage mapping
SPM: Spikelet pair meristem
SSSL: Single-segment substitution line

## References

[1] FAO (2020). Food and agriculture organization of the United Nations.

[2] Li H, Yang Q, Fan N, Zhang M, Zhai H, Ni Z (2017). Quantitative trait locus analysis of heterosis for plant height and ear height in an elite maize hybrid zhengdan 958 by design III. BMC Genet.

[3] Collard BCY, Mackill DJ (2008). Marker-assisted selection: an approach for precision plant breeding in the twenty-first century. Philos Trans R Soc B Biol Sci.

[4] Li C, Li Y, Sun B, Peng B, Liu C, Liu Z (2013). Quantitative trait loci mapping for yield components and kernel-related traits in multiple connected RIL populations in maize. Euphytica.

[5] Zhou Q, Wang PX, Cheng BJ (2014). Meta-analysis of QTL for ear row number in maize. J Maize Sci.

[6] Li M, Zhong W, Yang F, Zhang Z (2018). Genetic and molecular mechanisms of quantitative trait loci controlling maize inflorescence architecture. Plant Cell Physiol.

[7] Bommert P, Lunde C, Nardmann J, Vollbrecht E, Running M, Jackson D (2005). *thick tassel dwarf1* encodes a putative maize ortholog of the *Arabidopsis* CLAVATA1 leucine-rich repeat receptor-like kinase. Development.

[8] Vollbrecht E, Springer PS, Goh L, Buckler Iv ES, Martienssen R (2005). Architecture of floral branch systems in maize and related grasses. Nature.

[9] Bortiri E, Chuck G, Vollbrecht E, Rocheford T, Martienssen R, Hake S (2006). *ramosa2* encodes a LATERAL ORGAN BOUNDARY domain protein that determines the fate of stem cells in branch meristems of maize. Plant Cell.

[10] Bommert P, Nagasawa NS, Jackson D (2013). Quantitative variation in maize kernel row number is controlled by the FASCIATED EAR2 locus. Nat Genet.

[11] Liu L, Du Y, Shen X, Li M, Sun W, Huang J (2015). *KRN4* controls quantitative variation in maize kernel row number. PLoS Genet.

[12] Chuck GS, Brown PJ, Meeley R, Hake S (2014). Maize SBP-box transcription factors *unbranched2* and *unbranched3* affect yield traits by regulating the rate of lateral primordia initiation. Proc Natl Acad Sci U S A.

[13] Je BI, Gruel J, Lee YK, Bommert P, Arevalo ED, Eveland AL (2016). Signaling from maize organ primordia via FASCIATED EAR3 regulates stem cell proliferation and yield traits. Nat Genet.

[14] Je BI, Xu F, Wu Q, Liu L, Meeley R, Gallagher JP (2018). The CLAVATA receptor FASCIATED EAR2 responds to distinct CLE peptides by signaling through two downstream effectors. Elife.

[15] Trung KH, Tran QH, Bui NH, Tran TT, Luu KQ, Tran NTT (2020). A weak allele of *FASCIATED EAR 2* (*FEA2*) increases maize kernel row number (KRN) and yield in elite maize hybrids. Agronomy.

[16] Liu L, Gallagher J, Arevalo ED, Chen R, Skopelitis T, Wu Q (2021). Enhancing grain-yield-related traits by CRISPR-Cas9 promoter editing of maize CLE genes. Nat Plants.

[17] Wang J, Lin Z, Zhang X, Liu H, Zhou L, Zhong S (2019). *krn1*, a major quantitative trait locus for kernel row number in maize. New Phytol.

[18] Chen W, Chen L, Zhang X, Yang N, Guo J, Wang M (2022). Convergent selection of a WD40 protein that enhances grain yield in maize and rice. Science.

[19] Xiao Y, Liu H, Wu L, Warburton M, Yan J (2017). Genome-wide association studies in maize: praise and stargaze. Mol Plant.

[20] Wang J, Zhang X, Lin Z (2018). QTL mapping in a maize F_2_ population using genotyping-by-Sequencing and a modified fine-mapping strategy. Plant Sci.

[21] Sa KJ, Choi IY, Park JY, Choi JK, Ryu SH, Lee JK (2021). Mapping of QTL for agronomic traits using high-density SNPs with an RIL population in maize. Genes Genom.

[22] Zhang Y, Liang T, Chen M, Zhang Y, Wang T, Lin H (2019). Genetic dissection of stalk lodging-related traits using an IBM Syn10 DH population in maize across three environments (Zea mays L.). Mol Genet Genom.

[23] Li F, Jia HT, Liu L, Zhang CX, Liu ZJ, Zhang ZX (2014). Quantitative trait loci mapping for kernel row number using chromosome segment substitution lines in maize. Genet Mol Res.

[24] McMullen MD, Kresovich S, Villeda HS, Bradbury P, Li H, Sun Q (2009). Genetic properties of the maize nested association mapping population. Science.

[25] Gesteiro N, Cao A, Santiago R, Malvar RA, Butrón A (2021). Genomics of maize resistance to kernel contamination with fumonisins using a multiparental advanced generation InterCross maize population (MAGIC). BMC Plant Biol.

[26] Liu H-J, Wang X, Xiao Y, Luo J, Qiao F, Yang W (2020). CUBIC: an atlas of genetic architecture promises directed maize improvement. Genome Biol.

[27] Gage JL, Monier B, Giri A, Buckler ES (2020). Ten years of the maize nested association mapping population: impact, limitations, and future directions. Plant Cell.

[28] Nice LM, Steffenson BJ, Brown-Guedira GL, Akhunov ED, Liu C, Kono TJY (2016). Development and genetic characterization of an advanced backcross-nested association mapping (AB-NAM) population of wild × cultivated barley. Genetics.

[29] Bouchet S, Olatoye MO, Marla SR, Perumal R, Tesso T, Yu J (2017). Increased power to dissect adaptive traits in global sorghum diversity using a nested association mapping population. Genetics.

[30] Kidane YG, Gesesse CA, Hailemariam BN, Desta EA, Mengistu DK, Fadda C (2019). A large nested association mapping population for breeding and quantitative trait locus mapping in Ethiopian durum wheat. Plant Biotechnol J.

[31] Fragoso CA, Moreno M, Wang Z, Heffelfinger C, Arbelaez LJ, Aguirre JA (2017). Genetic architecture of a rice nested association mapping population. G3 Genes Genomes Genet.

[32] Li YX, Li C, Bradbury PJ, Liu X, Lu F, Romay CM (2016). Identification of genetic variants associated with maize flowering time using an extremely large multi-genetic background population. Plant J.

[33] Kump KL, Bradbury PJ, Wisser RJ, Buckler ES, Belcher AR, Oropeza-Rosas MA (2011). Genome-wide association study of quantitative resistance to southern leaf blight in the maize nested association mapping population. Nat Genet.

[34] Poland JA, Bradbury PJ, Buckler ES, Nelson RJ (2011). Genome-wide nested association mapping of quantitative resistance to northern leaf blight in maize. Proc Natl Acad Sci U S A.

[35] Cook JP, McMullen MD, Holland JB, Tian F, Bradbury P, Ross-Ibarra J (2012). Genetic architecture of maize kernel composition in the nested association mapping and inbred association panels. Plant Physiol.

[36] Zhao S, Li X, Song J, Li H, Zhao X, Zhang P (2022). Genetic dissection of maize plant architecture using a novel nested association mapping population. Plant Genome.

[37] Xiao Y, Tong H, Yang X, Xu S, Pan Q, Qiao F (2016). Genome-wide dissection of the maize ear genetic architecture using multiple populations. New Phytol.

[38] Chen J, Zeng B, Zhang M, Xie S, Wang G, Hauck A (2014). Dynamic transcriptome landscape of maize embryo and endosperm development. Plant Physiol.

[39] Calderón CI, Yandell BS, Doebley JF (2016). Fine mapping of a QTL associated with kernel row number on chromosome 1 of maize. PLoS ONE.

[40] Brown PJ, Upadyayula N, Mahone GS, Tian F, Bradbury PJ, Myles S (2011). Distinct genetic architectures for male and female inflorescence traits of maize. PLoS Genet.

[41] Liu L, Du Y, Huo D, Wang M, Shen X, Yue B (2015). Genetic architecture of maize kernel row number and whole genome prediction. Theor Appl Genet.

[42] Nie N, Ding X, Chen L, Wu X, An Y, Li C (2019). Characterization and fine mapping of *qkrnw4*, a major QTL controlling kernel row number in maize. Theor Appl Genet.

[43] Shen X, Zhao R, Liu L, Zhu C, Li M, Du H (2019). Identification of a candidate gene underlying *qKRN5b* for kernel row number in *Zea mays* L. Theor Appl Genet.

[44] Hufford MB, Xu X, van Heerwaarden J, Pyhäjärvi T, Chia JM, Cartwright RA (2012). Comparative population genomics of maize domestication and improvement. Nat Genet.

[45] Han X, Qin Y, Sandrine AMN, Qiu F (2020). Fine mapping of *qKRN8*, a QTL for maize kernel row number, and prediction of the candidate gene. Theor Appl Genet.

[46] An Y, Chen L, Li YX, Li C, Shi Y, Zhang D (2022). Fine mapping qKRN5.04 provides a functional gene negatively regulating maize kernel row number. Theor Appl Genet.

[47] Ibitoye DO, Akin-Idowu PE (2010). Marker-assisted-selection (MAS): a fast track to increase genetic gain in horticultural crop breeding. Afr J Biotechnol.

[48] Stirnimann CU, Petsalaki E, Russell RB, Müller CW (2010). WD40 proteins propel cellular networks. Trends Biochem Sci.

[49] Jain BP, Pandey S (2018). WD40 repeat proteins: signalling scaffold with diverse functions. Protein J.

[50] Wu Y, Li X, Xiang W, Zhu C, Lin Z, Wu Y (2012). Presence of tannins in sorghum grains is conditioned by different natural alleles of Tannin1. Proc Natl Acad Sci U S A.

[51] Wu Q, Xu F, Liu L, Char SN, Ding Y, Je BI (2020). The maize heterotrimeric G protein β subunit controls shoot meristem development and immune responses. Proc Natl Acad Sci U S A.

[52] Gallavotti A (2013). The role of auxin in shaping shoot architecture. J Exp Bot.

[53] Phillips KA, Skirpan AL, Liu X, Christensen A, Slewinski TL, Hudson C (2011). *vanishing tassel2* encodes a grass-specific tryptophan aminotransferase required for vegetative and reproductive development in maize. Plant Cell.

[54] Gallavotti A, Barazesh S, Malcomber S, Hall D, Jackson D, Schmidt RJ (2008). sparse inflorescence1 encodes a monocot-specific YUCCA-like gene required for vegetative and reproductive development in maize. Proc Natl Acad Sci U S A.

[55] Koizumi K, Hayashi T, Wu S, Gallagher KL (2012). The SHORT-ROOT protein acts as a mobile, dose-dependent signal in patterning the ground tissue. Proc Natl Acad Sci U S A.

[56] Zhang ZL, Ogawa M, Fleet CM, Zentella R, Hu J, Heo JO (2011). SCARECROW-LIKE 3 promotes gibberellin signaling by antagonizing master growth repressor DELLA in *Arabidopsis*. Proc Natl Acad Sci U S A.

[57] Sun L, Li X, Fu Y, Zhu Z, Tan L, Liu F (2013). *GS6*, a member of the GRAS gene family, negatively regulates grain size in rice. J Integr Plant Biol.

[58] Torres-Galea P, Hirtreiter B, Bolle C (2013). Two GRAS proteins, SCARECROW-LIKE21 and PHYTOCHROME A SIGNAL TRANSDUCTION1, function cooperatively in phytochrome a signal transduction. Plant Physiol.

[59] Cai H, Chen Y, Zhang M, Cai R, Cheng B, Ma Q (2017). A novel GRAS transcription factor, ZmGRAS20, regulates starch biosynthesis in rice endosperm. Physiol Mol Biol Plants.

[60] Li Y, Ma S, Zhao Q, Lv D, Wang B, Xiao K (2021). ZmGRAS11, transactivated by *Opaque2*, positively regulates kernel size in maize. J Integr Plant Biol.

[61] R Core Team (2015). R: a language and environment for statistical computing.

[62] Broman KW, Wu H, Sen S, Churchill GA (2003). R/qtl: QTL mapping in experimental crosses. Bioinformatics.

[63] Kosambi DD (1944). The estimation of map distances from recombination values. Ann Eugen.

[64] Chen Q, Yang CJ, York AM, Xue W, Daskalska LL, DeValk CA (2019). TeoNAM: a nested association mapping population for domestication and agronomic trait analysis in maize. Genetics.

[65] Meng L, Li H, Zhang L, Wang J (2015). QTL IciMapping: integrated software for genetic linkage map construction and quantitative trait locus mapping in biparental populations. Crop J.

[66] Chakraborty R, Weiss KM (1988). Admixture as a tool for finding linked genes and detecting that difference from allelic association between loci. Proc Natl Acad Sci U S A.

[67] Yang J, Lee SH, Goddard ME, Visscher PM (2011). GCTA: a tool for genome-wide complex trait analysis. Am J Hum Genet.

[68] Liu X, Huang M, Fan B, Buckler ES, Zhang Z (2016). Iterative usage of fixed and random effect models for powerful and efficient genome-wide association studies. PLoS Genet.

[69] Li X, Wang M, Zhang R, Fang H, Fu X, Yang X (2022). Genetic architecture of embryo size and related traits in maize. Crop J.

[70] Kim D, Langmead B, Salzberg SL (2015). HISAT: a fast spliced aligner with low memory requirements. Nat Methods.

