# Supplementary material for: Identification of two new QTLs of maize (Zea mays L.) underlying kernel row number using the HNAU-NAM1 population
Source: BMC Genomics. 2022 Aug 15;23:593. doi: 10.1186/s12864-022-08793-1 (PMC9380338; doi:10.1186/s12864-022-08793-1)
Supplement: Supplementary file 2 — Additional file 2: Fig. S1 Genetic linkage maps in the 12 subpopulations of HNAU-NAM1. Red: GEMS41 genotype; green: genotype of other parents; blue: heterozygote. The ordinate represents the number of RILs. Fig. S2 Expression profiles of the genes located in the QTL regions qKRN4.2 (A) and qKRN9.1 (B). The expression values were collected from a public database (www.maizegdb.org) and normalized by the logarithm of fragments per kilobase of exon model per million mapped fragments (Log2(RPKM+1)). Each column represents a tissue, and the rows indicate genes expressed in the ear [file 12864_2022_8793_MOESM2_ESM.docx]

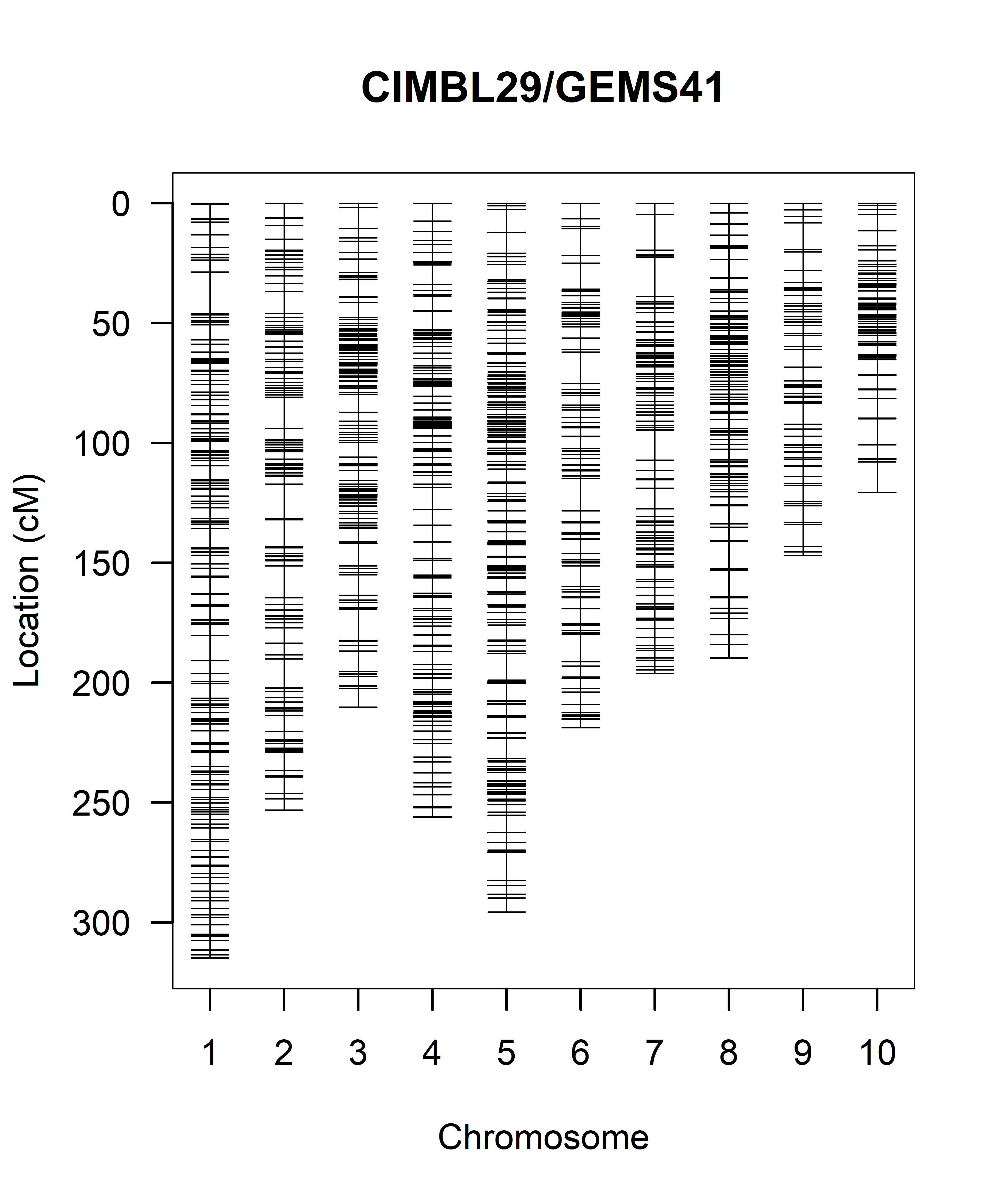

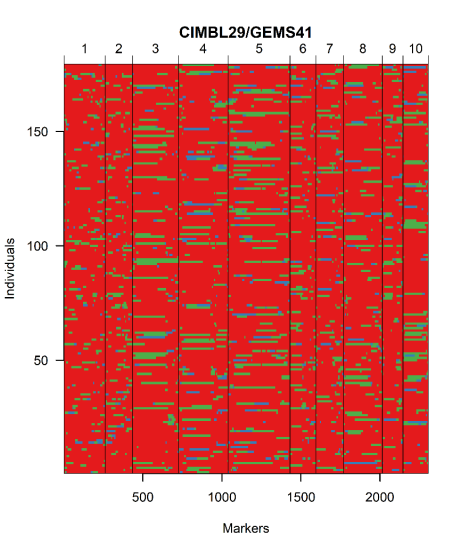


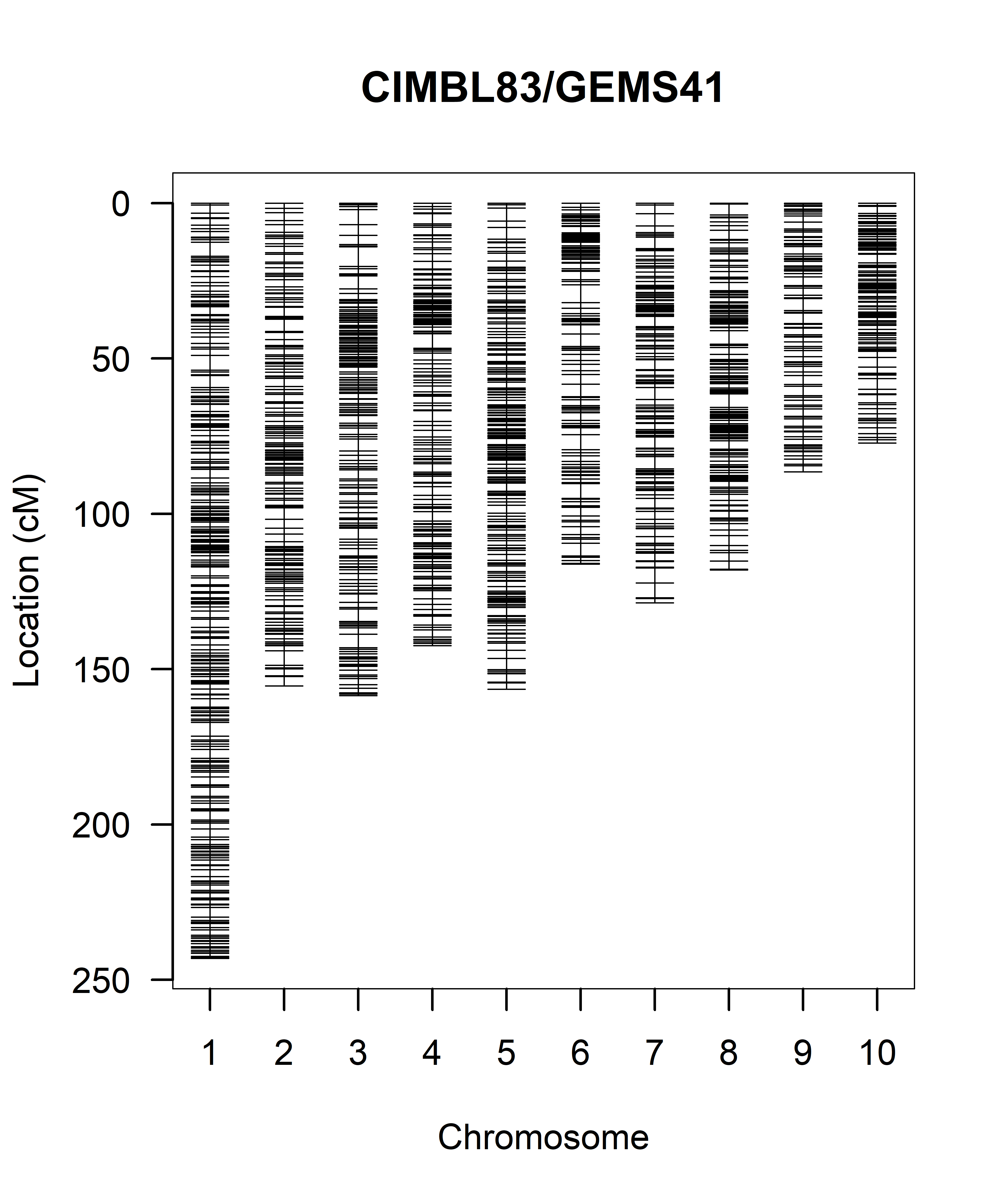

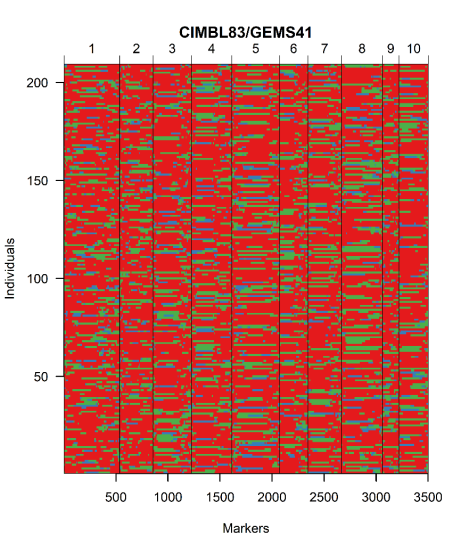


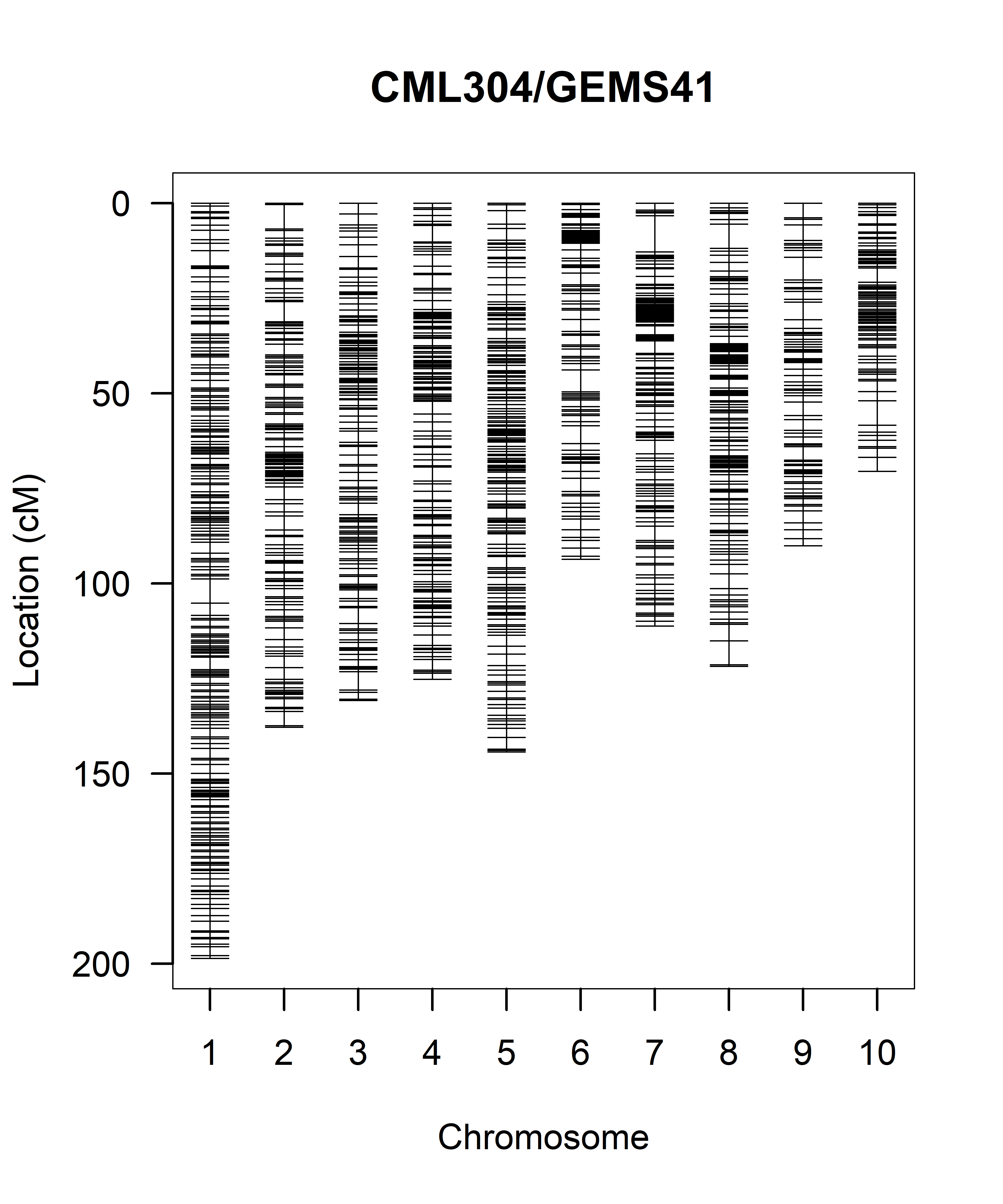

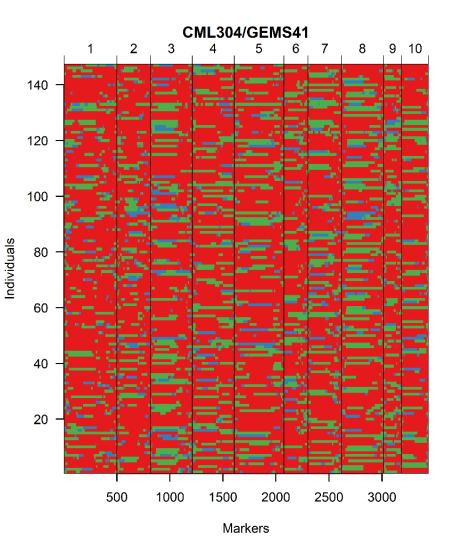


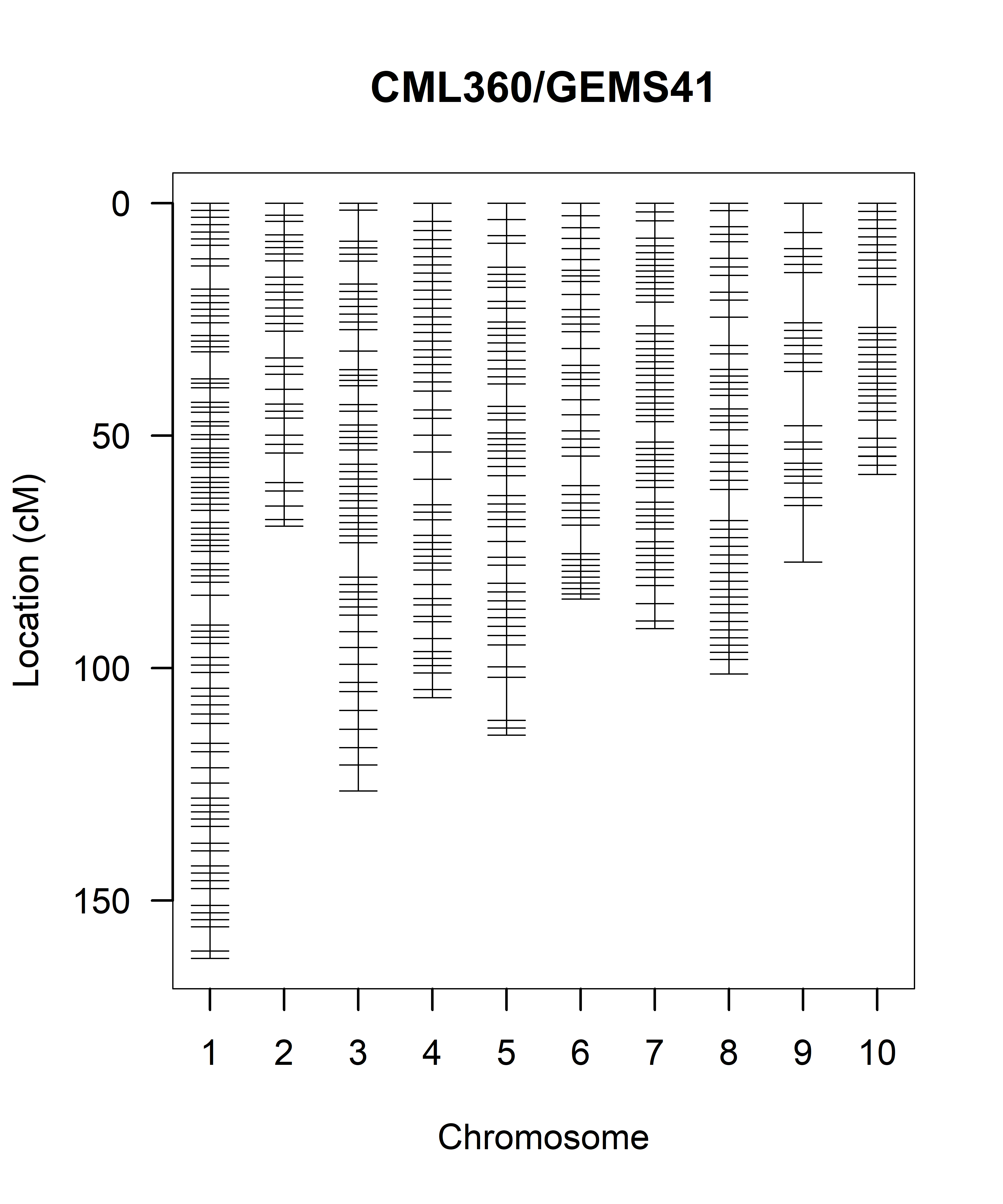

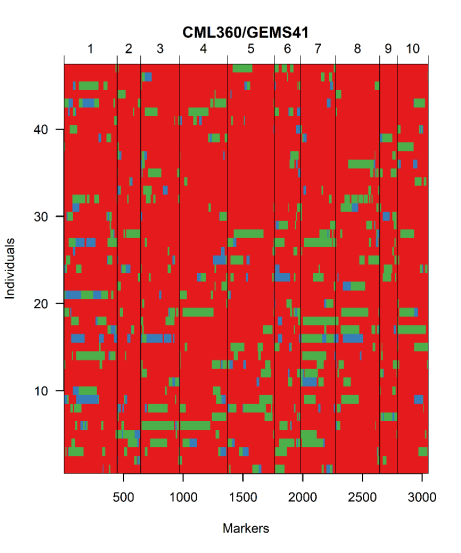


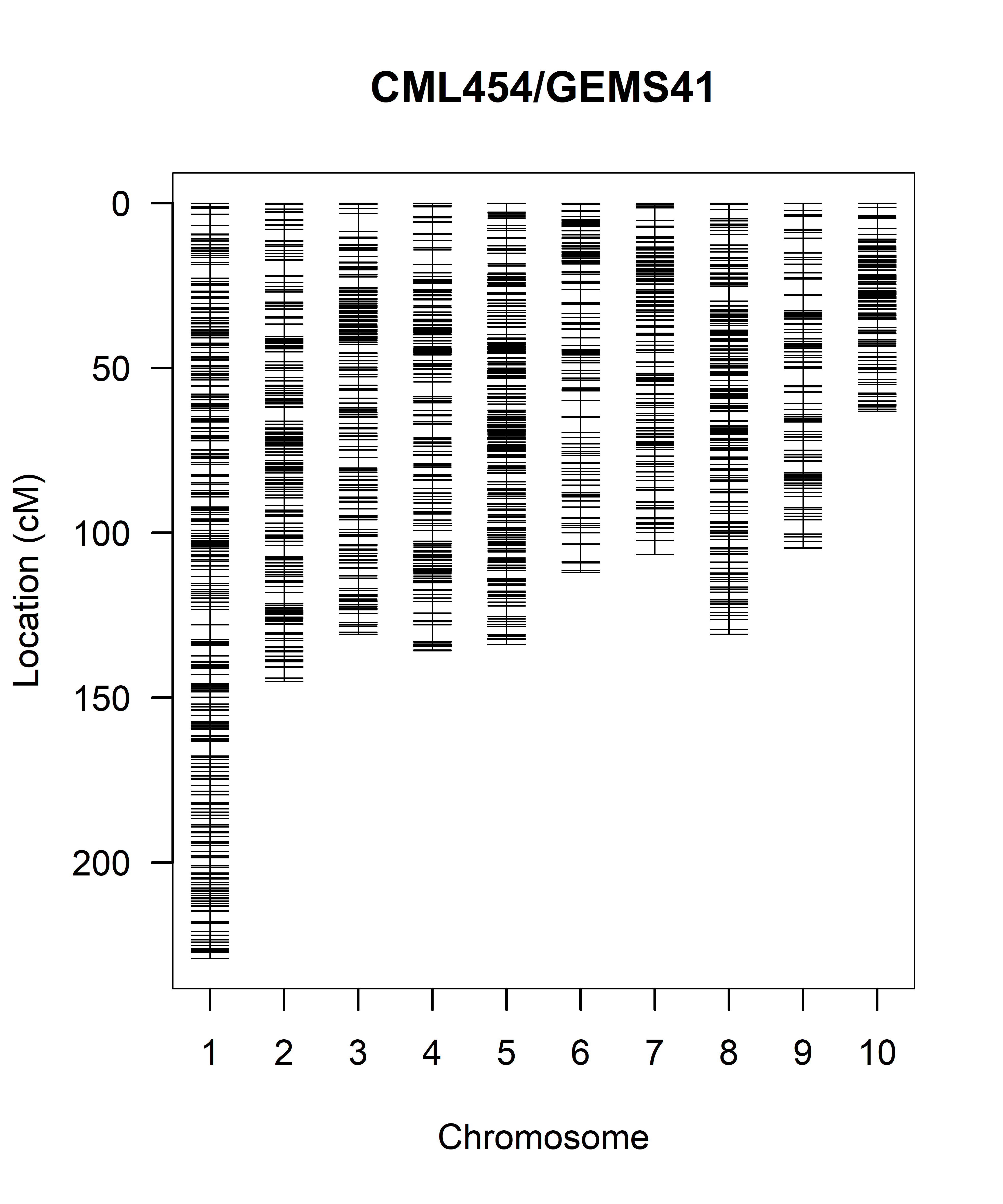

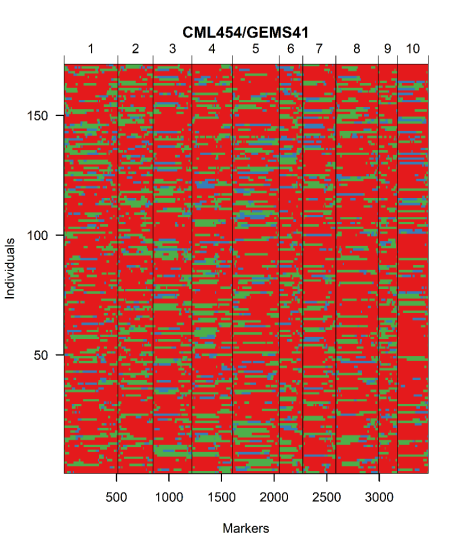


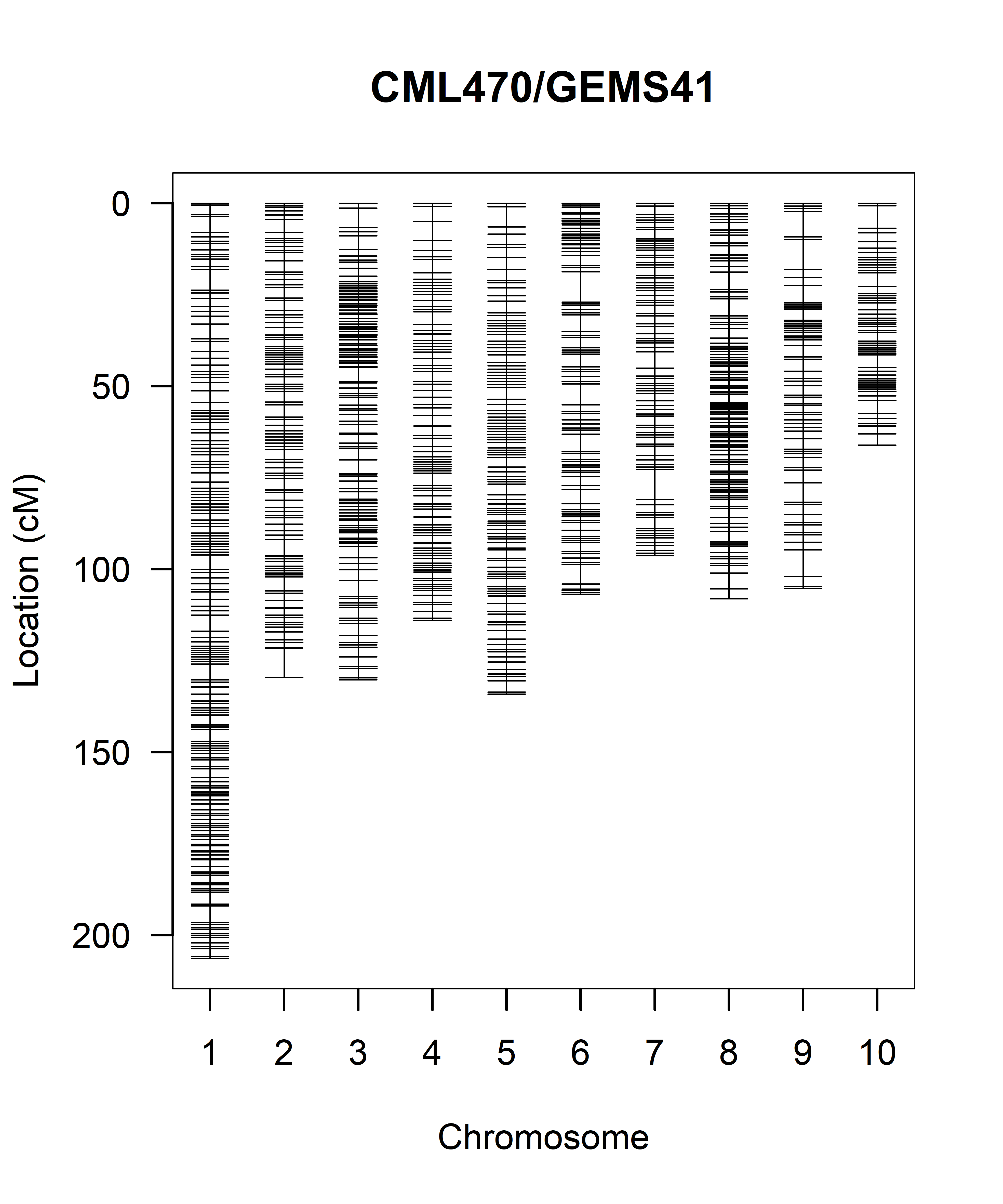

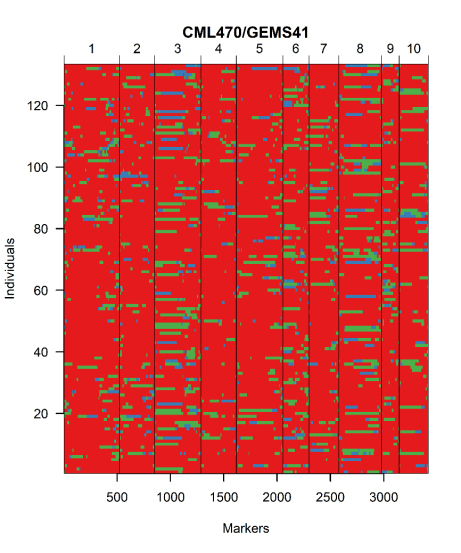


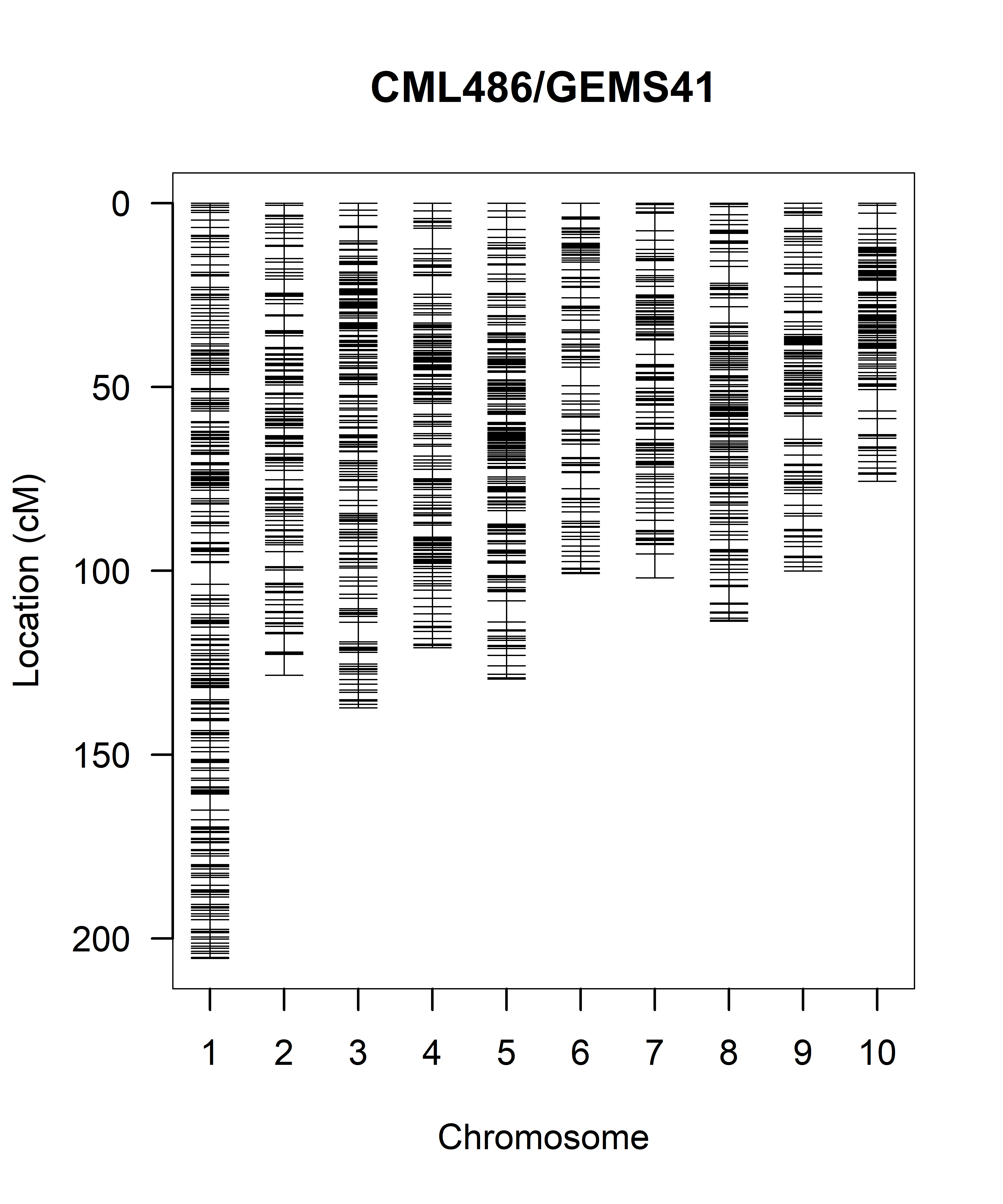

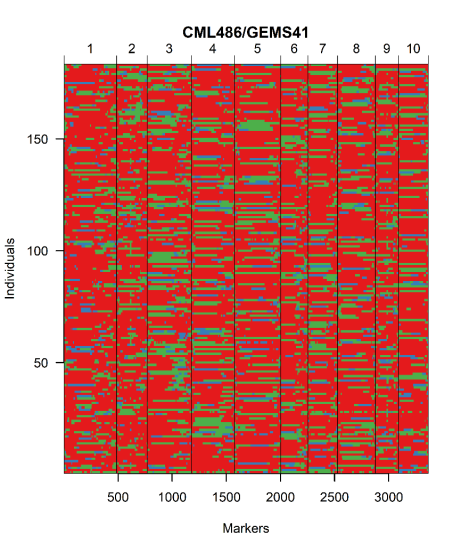


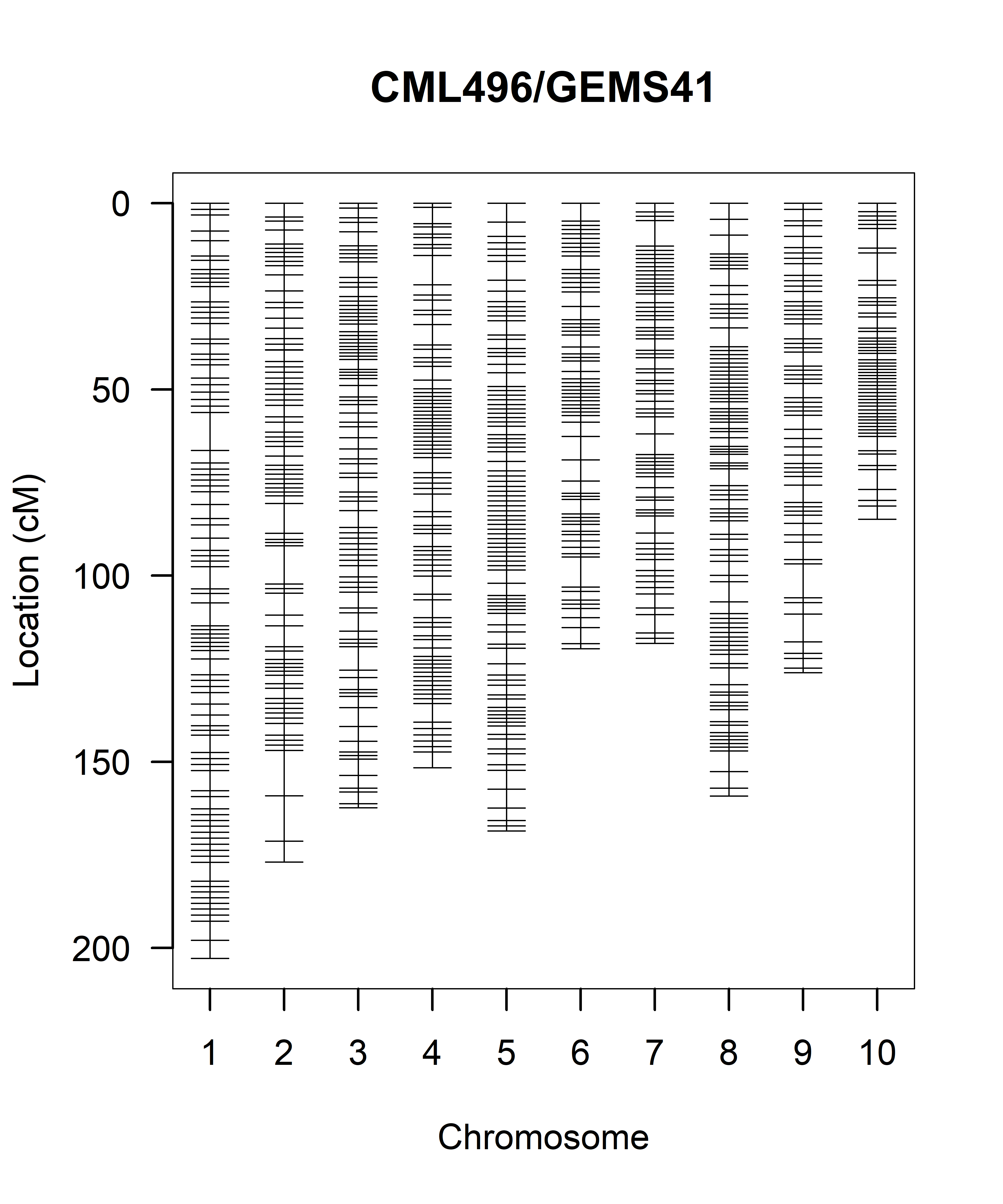

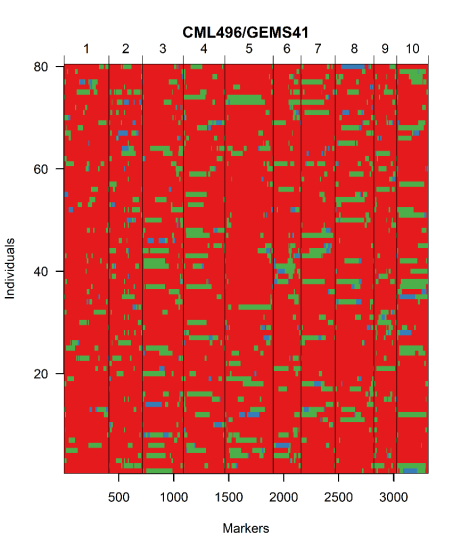


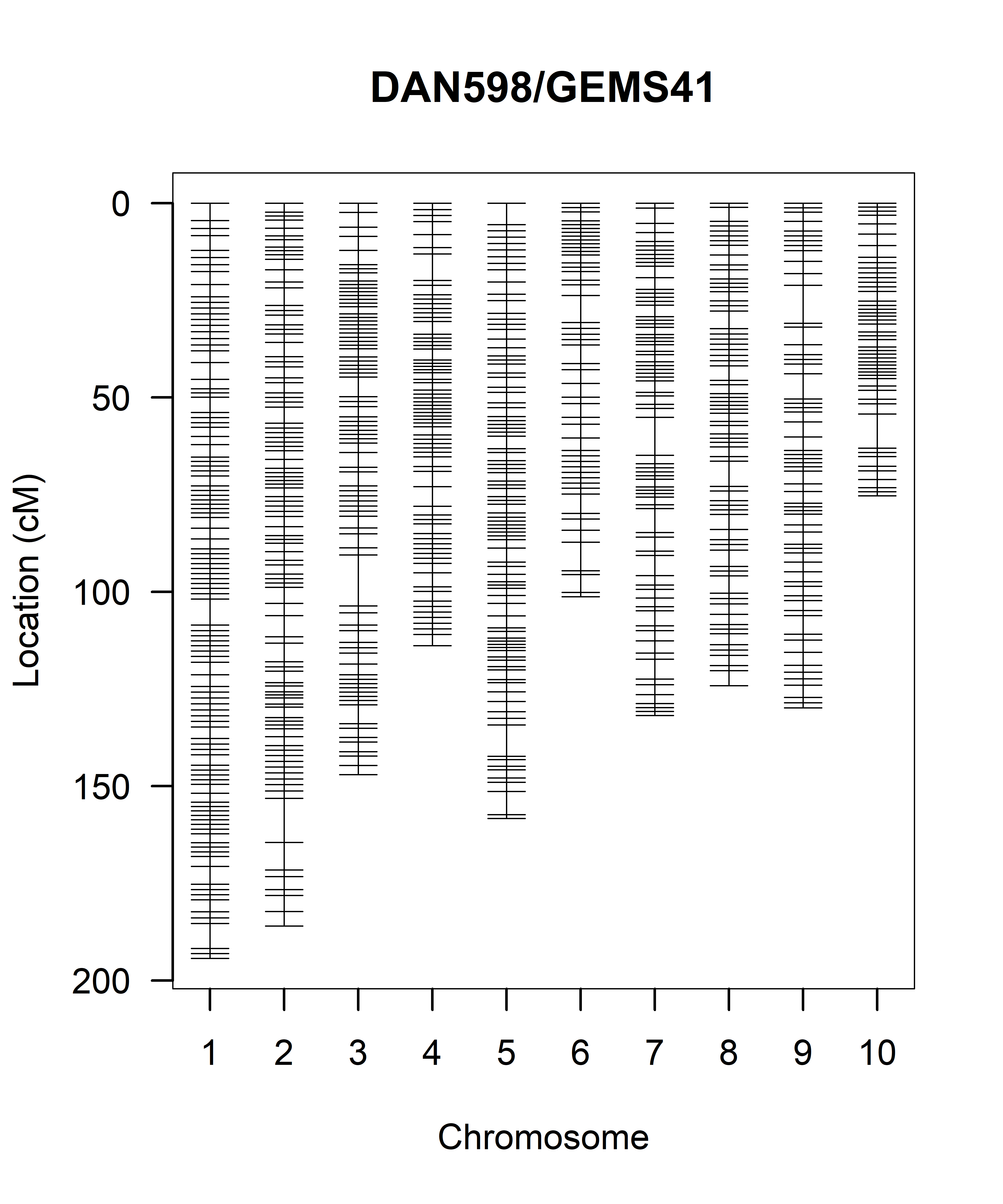

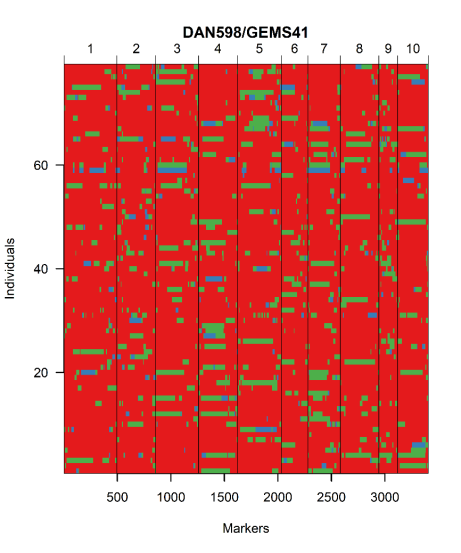


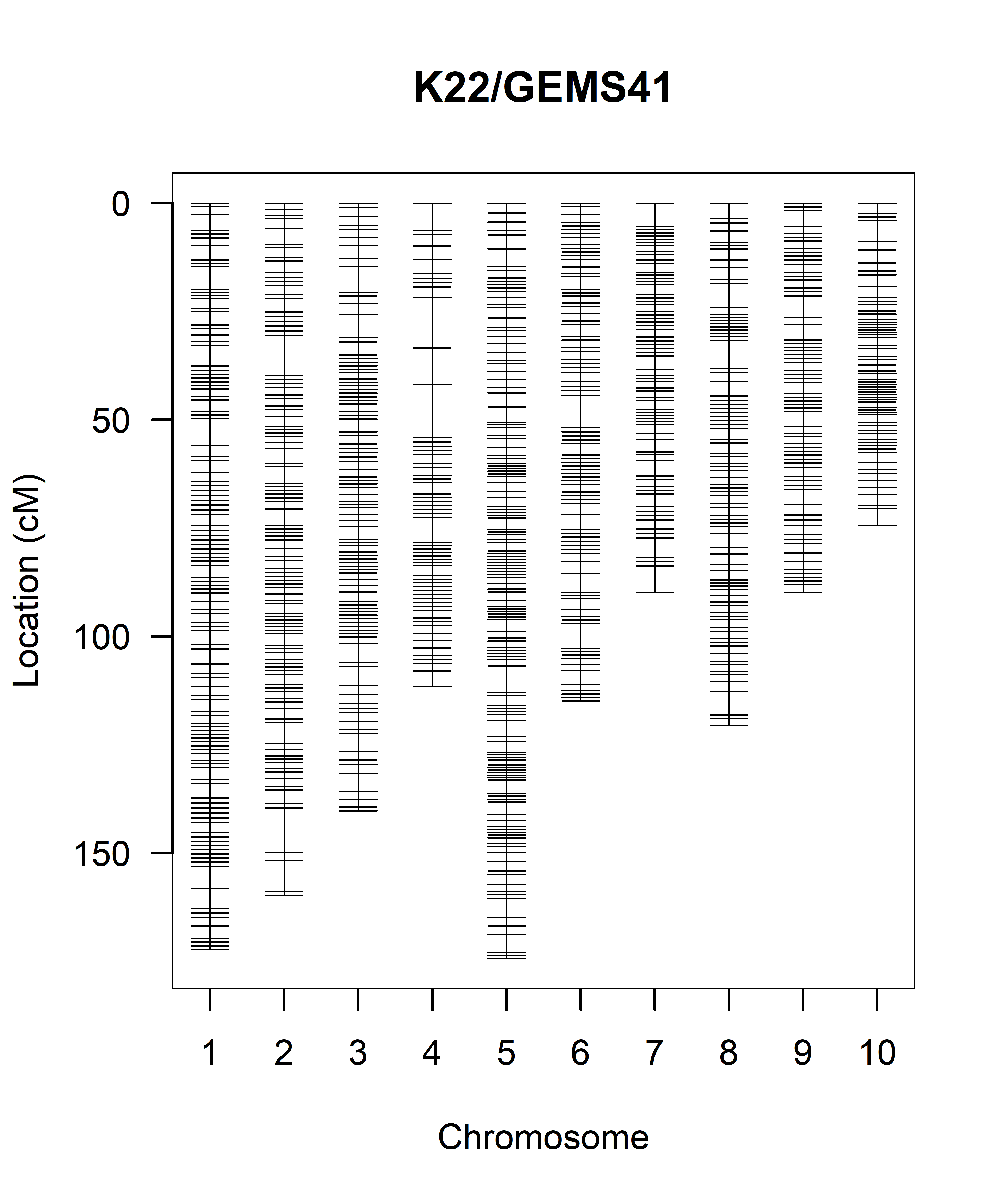

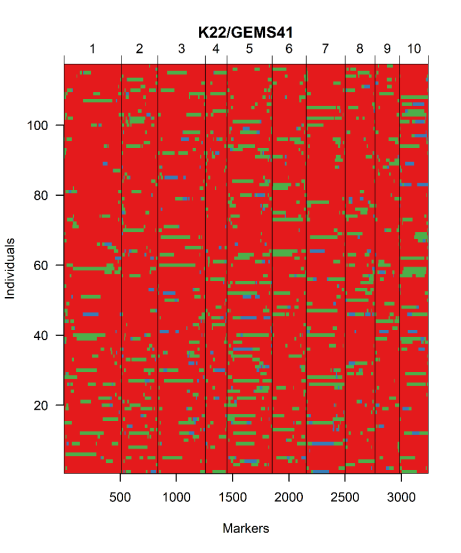


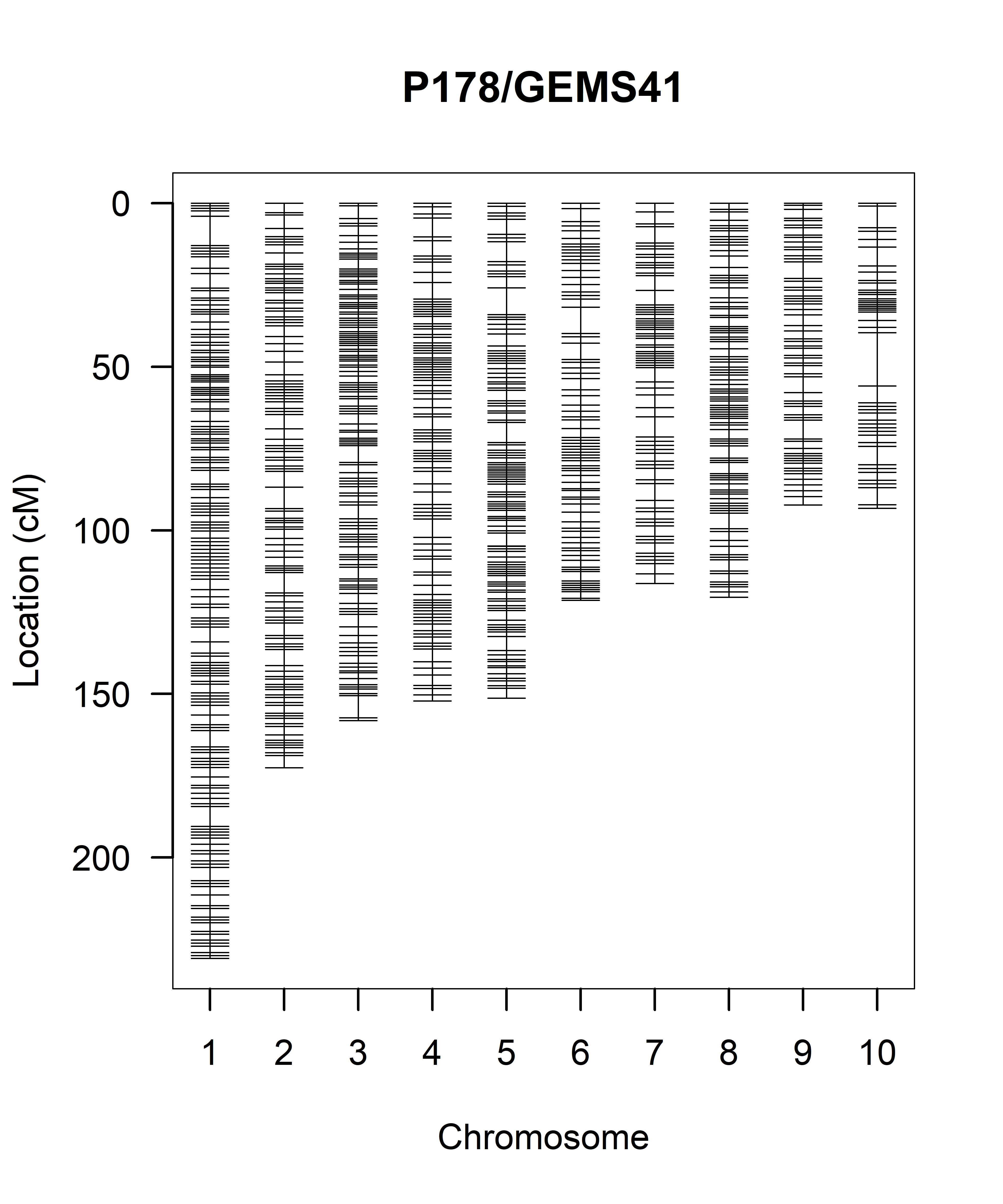

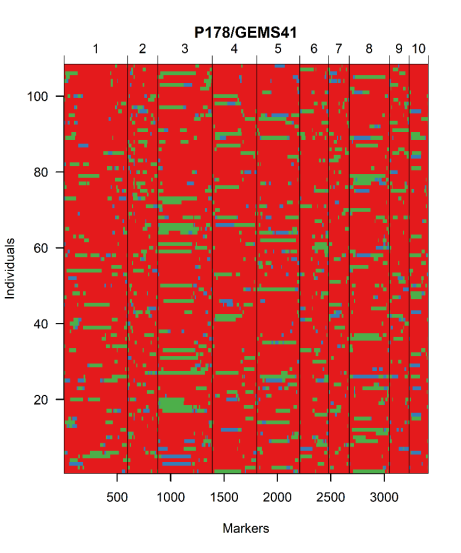


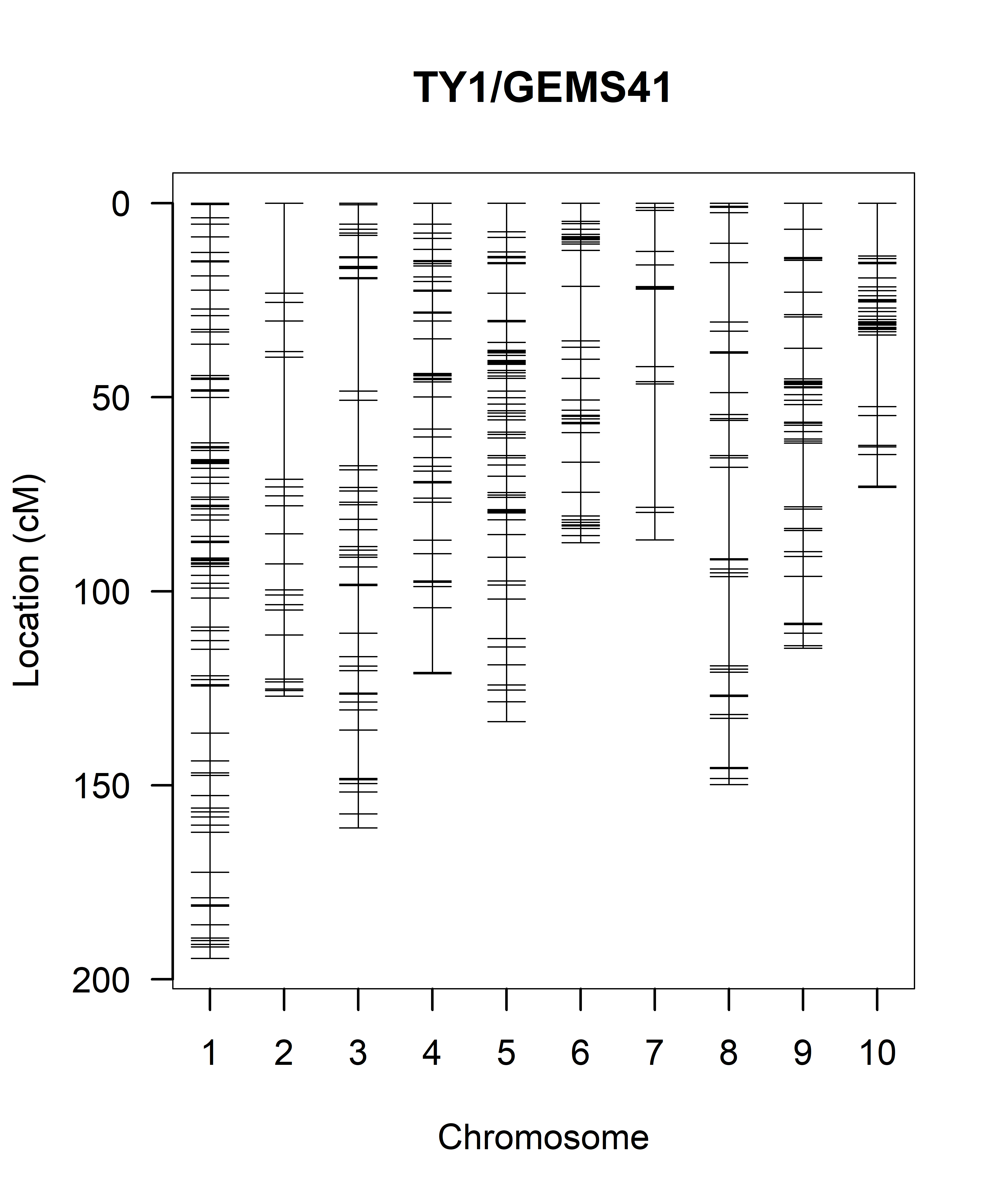

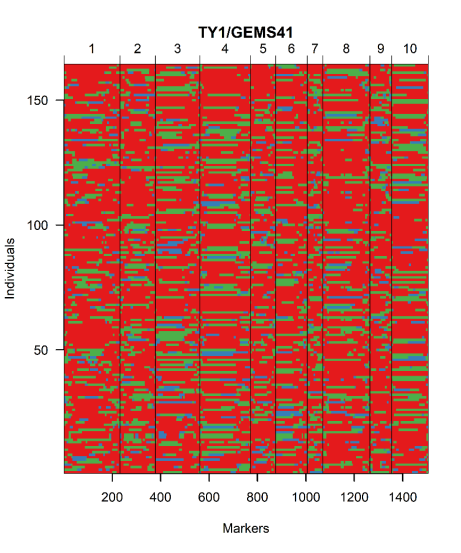


**Fig. S1** Genetic linkage maps in the 12 subpopulations of HNAU-NAM1. Red: GEMS41 genotype; green: genotype of other parents; blue: heterozygote. The ordinate represents the number of RILs.


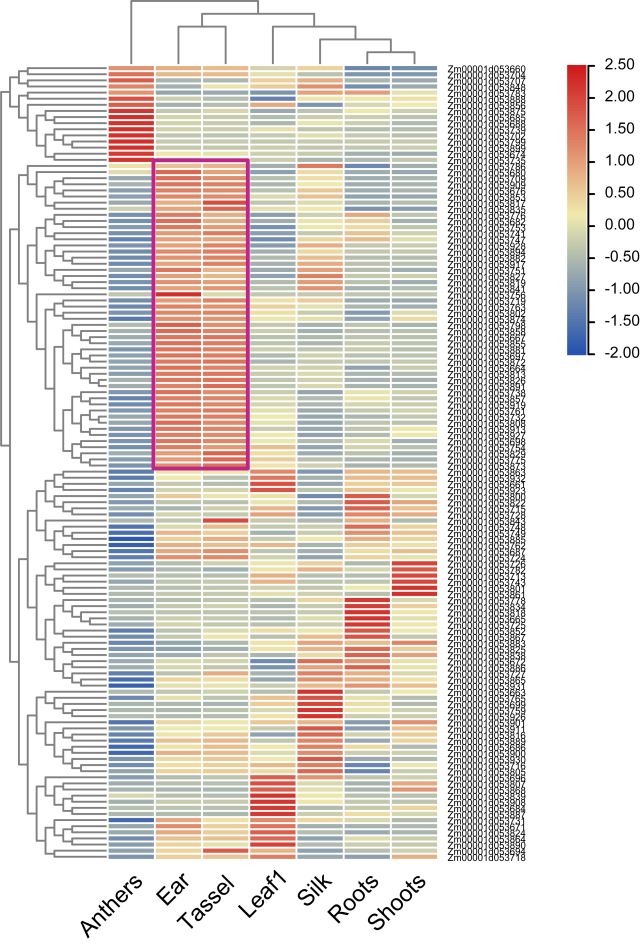

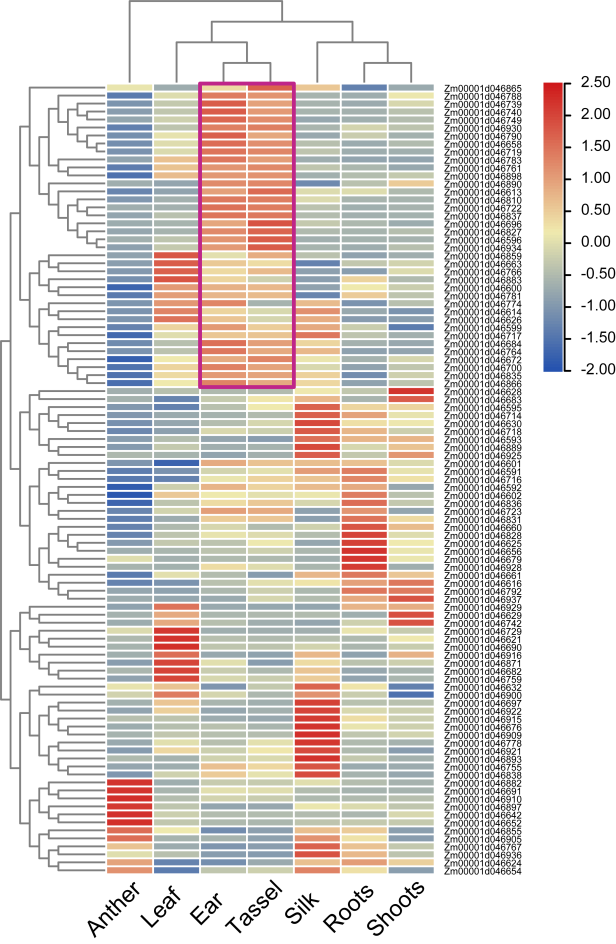


A

B

**Fig. S2** Expression profile of the genes located in the QTL regions *qKRN4.2* (A) and *qKRN9.1* (B).

The expression values were collected from a pubic database ([www.maizegdb.org](http://www.maizegdb.org)), and normalized by the logarithm of fragments per kilobase of exon model per million mapped fragments Log_2_(RPKM+1). Each column represents a tissue, and the rows indicate genes expressed in the ear.
